# Blended mobile health and wellness coaching enhances student engagement in mental health care

**DOI:** 10.20935/mhealthwellb8298

**Published:** 2026-05-25

**Authors:** Michael Reifman, Xiao Cao, Michelle L. Rozwadowski, Jaeryeong Kim, Maxwell Kluge, Uma L. Subrayan, Marsha Benz, Amanda Reis, Jevon Moore, Kerby Shedden, Sung Won Choi

**Affiliations:** 1 Department of Pediatrics, Medical School, University of Michigan, Ann Arbor, MI, USA.; 2 Wolverine Wellness, Division of Student Life, University of Michigan, Ann Arbor, MI, USA.; 3 Department of Statistics, College of Literature, Sciences, and the Arts, University of Michigan, Ann Arbor, MI, USA.

**Keywords:** mental health, psychological well-being, mHealth, mobile health, University Health Services

## Abstract

**Introduction:**

Mental health challenges are common among college students, highlighting the need for scalable approaches that aim to reduce distress and support well-being. mHealth tools may complement campus services, though sustained engagement may be difficult. This study sought to discover how a blended intervention combining an mHealth positive psychology intervention (PPI) app with optional wellness coaching might impact student well-being.

**Materials and methods:**

In this single-arm pilot study, 28 students at a public university were given access to a PPI app (Roadmap 2.0) with mood tracking, a Fitbit^®^ wearable device, and optional wellness coaching. Data sources included PROMIS^®^ surveys at baseline and monthly follow-ups, daily mood ratings, app engagement logs, wearable-derived activity metrics, coaching attendance, and optional exit interviews. Analyses were descriptive and exploratory.

**Results:**

From baseline to exit, participants showed descriptive increases in PROMIS^®^ global mental health and positive affect and decreases in depression, anxiety, fatigue, and anger. App engagement declined over time. In exploratory models, app engagement was lower among participants reporting greater psychosocial resources or support. Mood ratings were higher in the days following PPI activity completion, and PPI activity users were observed to have higher mood ratings over time. Interviews supported perceived app–coaching synergy and identified barriers to sustained engagement.

**Conclusions:**

A blended PPI mHealth app plus wellness coaching appears feasible in a real-world college setting and was associated with favorable descriptive trends in mental health and well-being outcomes. Controlled studies are needed to evaluate efficacy and assess app versus coaching contributions.

## 1. Introduction

### 1.1. College students and mental health

College students are among the populations most vulnerable to mental health problems, with rates of depression and anxiety rising significantly over the last decade. The American College Health Association reports that academic pressure and career challenges contribute to moderate or high perceived stress among students [1]. According to the Healthy Minds Network 2024 report, 77% of students cited that emotional or mental difficulties negatively affected their academic performance on at least one day in the past four weeks [2].

In 2024, 38% of U.S. college students reported moderate or severe depressive symptoms (Patient Health Questionnaire-9 ≥10) and 34% reported moderate or severe anxiety (Generalized Anxiety Disorder-7 ≥10) [2]. There has also been a 17% decrease in positive mental health between 2012 and 2021 [2,3]. Together, these trends highlight an urgent need for intervention in this population, as nearly 50% of students reported a current perceived need of help for emotional or mental health issues [2].

### 1.2. Theoretical and conceptual framing: positive psychology, well-being, and ill-being

The theoretical and conceptual framing of this study was grounded in positive psychology, including Seligman’s model of flourishing, which conceptualized well-being as the active cultivation of core resources, Positive Emotion, Engagement, Relationships, Meaning, and Accomplishment (PERMA), rather than solely the absence of mental illness [4]. Importantly, this framing is aligned with the two continua model, which posits that poor mental health (“ill-being”) and well-being represent related but distinct dimensions, such that reductions in distress do not necessarily produce corresponding increases in well-being [5]. This may be relevant in college settings, where promoting student thriving may require assessing multidimensional well-being alongside symptoms [6]. In support of this approach, Kern et al. showed that the PERMA framework can be applied to students as a multidimensional measurement model, reinforcing the value of assessing well-being across multiple domains relevant to functioning and flourishing [7]. Accordingly, in the present study, as well as in alignment with our previous work in college students [8,9], we evaluate outcomes spanning both ill-being (e.g., anxiety, depression, and related distress domains) and well-being, including health-related quality of life (HRQOL) and role/social functioning, using validated self-reported measures to capture a more complete picture of student mental health.

### 1.3. Positive psychology interventions and mobile health

Positive psychology interventions (PPIs) are structured strategies designed to increase well-being through well-studied constructs, such as positive reflection, gratitude, and savoring [4]. Simple PPI activities that enhance positive thoughts, emotions, and behaviors have shown benefits in prior studies and may be scalable [10]. PPIs have been used in students to support engagement, learning, and well-being [11], as well as in individuals managing chronic disease and stress-related conditions [12]. An economic evaluation of a PPI designed to foster positive emotions, stimulate positive functioning, and reduce depressive symptoms found improved cost-effectiveness for individuals randomized to the PPI arm compared with a waitlisted usual care group [13]. Collectively, this evidence supports PPIs as a plausible, skills-based approach to strengthening well-being in college students.

Mobile health (mHealth) delivery can expand the reach of PPIs by enabling self-paced practice and frequent “in-the-moment” use. While gaps remain in understanding which app-based approaches work best and for whom [14], mental health apps are increasingly used, including among college students [15]. As of 2024, 91% of adults in the U.S. report owning a smartphone [16], suggesting that mHealth is a feasible and accessible delivery platform. Roadmap 2.0 is one such mHealth platform that delivers PPIs through interactive activities and self-monitoring features [9]. Well-designed studies using PPI activities have shown moderate effects on reducing depression and anxiety [17]. Roadmap 2.0 activities that prompt users to reflect on gratitude have been correlated with reductions in anxiety and depression among students [9]. However, a persistent challenge for digital mental health interventions is sustaining engagement over time and ensuring that activities translate into meaningful behavior change in daily life.

### 1.4. Wellness coaching

Wellness coaching is a participant-centered approach in which individuals set goals and actively engage with a trained professional who supports behavior change processes. Coaches do not provide clinical care; they collaborate with participants to develop skills and routines that support desired changes in mental and physical well-being, emphasizing human connection and accountability. Wolverine Wellness is a free service offered by the University of Michigan (U-M) to all U-M students, faculty, and staff [18].

### 1.5. Justification for the blended approach and mixed-methods evaluation

Several studies have examined the effect of digital interventions and wellness coaching on this college population, but these interventions have only ever been deployed separately from one another [9,14-16]. In the present study, a central justification for the combined approach is that mHealth PPIs and wellness coaching offer complementary strengths while also addressing key limitations when delivered alone. App-based PPIs, like Roadmap 2.0, can be scalable, accessible, and conducive to skills practice, but digital interventions commonly face declining engagement, limited personalization, and challenges integrating activities into users’ daily contexts [8,9]. In contrast, human coaching may provide goal-setting and accountability that could support engagement and facilitate the application of PPI skills. Thus, we designed a blended intervention, Roadmap 2.0 plus wellness coaching, leveraging the resources available at the U-M, to promote adherence and sustained PPI skills.

### 1.6. Main aims of the current pilot study

Building on our prior work [9] and the literature [19], we conducted a prospective single-arm pilot study combining the Roadmap 2.0 app with optional wellness coaching over one academic semester. The primary aim of this paper is descriptive: to report pilot findings and participant experiences with a blended mHealth and coaching approach in a real-world college setting. Consistent with a pilot design, the study was not intended for confirmatory hypothesis testing. Instead, we sought to characterize the following: (1) engagement with the blended intervention (app use, coaching session attendance, and wearable-derived outputs); (2) descriptive changes over time in self-reported outcomes and physical activity; (3) participant perspectives on perceived value, barriers, and opportunities for improvement based on qualitative interviews. These exploratory findings are intended to inform intervention refinement and the design of a subsequent randomized study.

## 2. Materials and methods

### 2.1. Study site

This pilot study was conducted at the U-M, a large, public university in Ann Arbor, MI, USA. Study activities were conducted with a combination of in-person and online contact.

### 2.2. Study design and participants

This study was a single-arm pilot conducted over one academic semester using a convergent mixed-methods design with quantitative and qualitative data collection. The trial was registered on ClinicalTrials.gov (NCT05736445).

**Figure 1.**
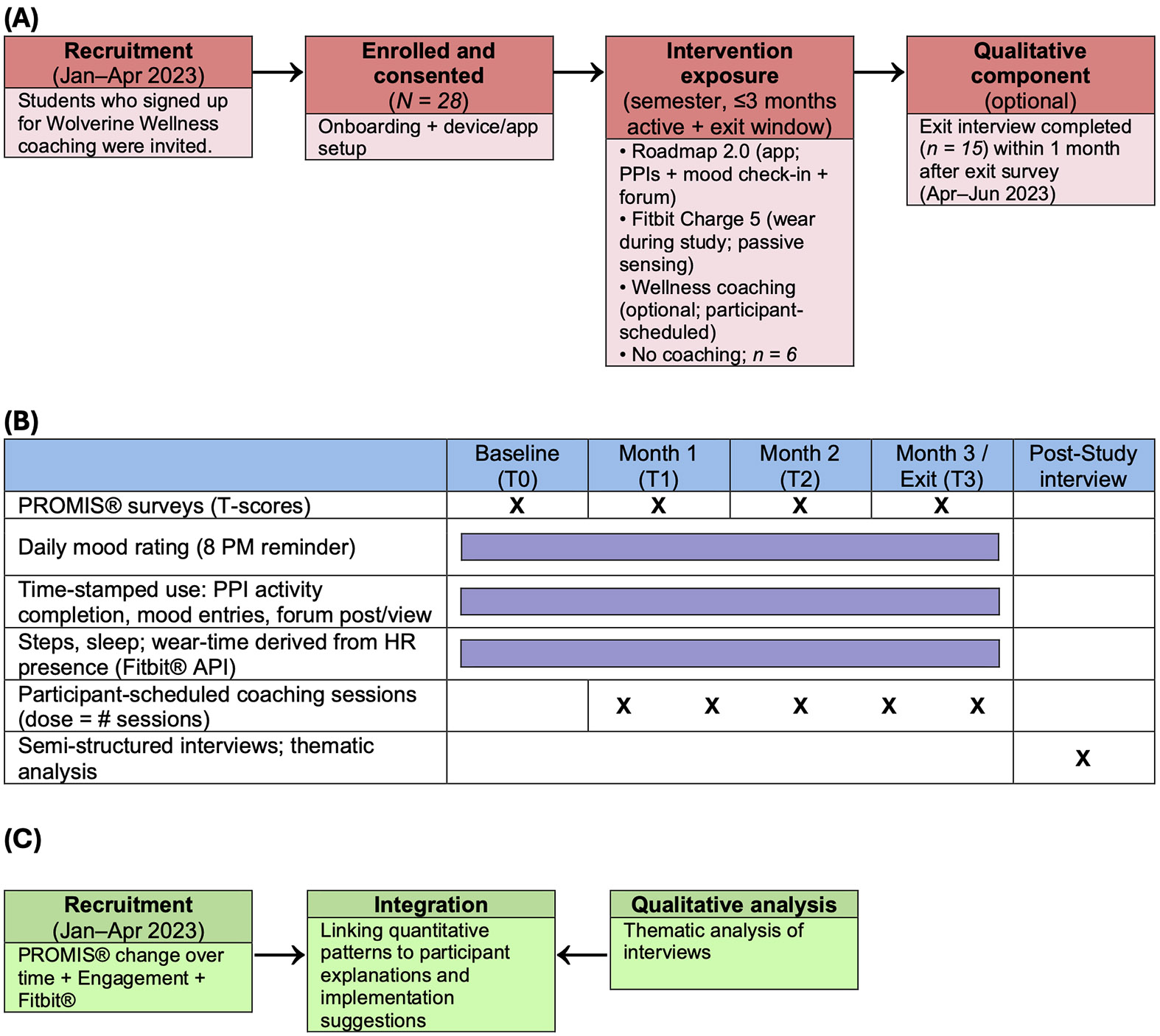
Study design and timeline. (**A**) Participant flow and intervention components. Recruitment occurred January–April 2023 among students who signed up for Wolverine Wellness coaching. Enrolled and consenting participants (*N* = 28) completed onboarding and device/app setup and received access to the Roadmap 2.0 positive psychology intervention (PPI) app (PPI activities, daily mood check-in, forum) and a Fitbit^®^ Charge 5 wearable for passive monitoring of activity and sleep during the study period (semester, ≤3 months active + exit window). Optional wellness coaching was offered and scheduled by participants (*n* = 22 attended ≥1 session; *n* = 6 received no coaching). An optional qualitative component included an exit interview completed within 1 month after the exit survey (*n* = 15; April–June 2023). Interview and coaching subgroups are subsets of the total enrolled cohort (*N* = 28; overlapping), not additional participants. (**B**) Data collection timeline. Quantitative outcomes included PROMIS^®^ health-related quality of life (HRQOL) surveys (T-scores) administered at baseline (T0), Month 1 (T1), Month 2 (T2), and Month 3/Exit (T3). The “X” represents a discrete instance within that time frame, whereas the shaded bar represents a continuous collection of data. (T3); daily mood ratings (8 PM reminder); time-stamped app engagement logs (PPI activity completion, mood entries, forum posting/viewing) throughout the study; and wearable-derived metrics (steps, sleep, wear-time derived from heart rate presence via Fitbit^®^ API) throughout the study. Participant-scheduled coaching sessions occurred during the intervention period. The dose equaled the # (number) of sessions. Qualitative data consisted of optional semi-structured post-study exit interviews conducted within one month after T3, followed by thematic analysis. (**C**) Convergent mixed-methods integration. Quantitative patterns (PROMIS^®^ change over time, engagement, and Fitbit^®^ metrics) were linked with qualitative themes from interviews to contextualize participant experiences and generate implementation suggestions.

As shown in Figure 1, the intervention included: (1) use of the Roadmap 2.0 app (Supplementary materials and Figure S3); (2) passive monitoring using a Fitbit^®^ Charge 5 (Google LLC, Mountain View, CA, USA) wearable linked via the Fitbit^®^ API; (3) optional wellness coaching delivered in-person or virtually and scheduled by participants. Quantitative outcomes included PROMIS^®^ HRQOL surveys administered at baseline (T0), Month 1 (T1), Month 2 (T2), and exit/Month 3 (T3), along with daily mood ratings, app engagement logs, and wearable-derived activity metrics collected throughout the study period. Qualitative data consisted of optional semi-structured interviews conducted within one month after the exit survey.

### 2.3. Eligibility

Participants were eligible if they were ≥18 years old, were a full-time undergraduate or graduate at U-M during winter 2023, had a United States (US) mailing address, could read/speak English, owned a smartphone, and could provide informed consent.

### 2.4. Recruitment and consent

Recruitment occurred from January to April 2023 through Wolverine Wellness (University Health Service), where students signing up for coaching were invited to participate [18]. All participants completed IRBMED-approved electronic consent via airSlate’s SignNow, Brookline, MA, USA. After consent, the study coordinator contacted participants to schedule onboarding.

### 2.5. Compensation

Participants received a Fitbit^®^ Charge 5 device (to wear during the study and keep). Additional compensation included: $10 for completion of the baseline survey (T0); $5 each for T1, T2, and T3 surveys (total $15); $10 for completing all four surveys; $10 for wearing the Fitbit^®^; $10 per attended coaching sessions (up to four sessions; $40 maximum); and $15 for completing the exit interview, which could be combined with a fifth coaching session.

### 2.6. Intervention components and data sources

#### 2.6.1. Roadmap 2.0 app and in-app mood tracking

During onboarding, participants installed Roadmap 2.0 and Fitbit^®^ apps and were provided with an access code to activate Roadmap 2.0 and link it to their Fitbit^®^ account. The Roadmap 2.0 app includes eight PPI activities, chat forums, and graphs summarizing mood, sleep, and step counts, as previously described [9]. Participants were instructed to use Roadmap 2.0 freely. Participants were also prompted via push notification to record a daily mood rating (1–10; 1 = worst, 10 = best), with a reminder sent via push notification to their smartphones at 8 PM.

#### 2.6.2. Fitbit^®^ wearable monitoring

Participants wore a Fitbit^®^ Charge 5 throughout the study period. Wearable-derived activity and sleep metrics were obtained via the Fitbit^®^ API, as described below (see “Fitbit^®^ wear-time”).

#### 2.6.3. Wellness coaching

Participants could schedule wellness coaching sessions at their preferred frequency (up to five sessions permitted). Coaching was delivered in-person or virtually by Wolverine Wellness coaches and incorporated a positive psychology-informed approach focused on goal planning, cultivating positive emotions, and directing participants to Roadmap’s PPIs when applicable.

### 2.7. Quantitative measures

#### 2.7.1. PROMIS^®^ surveys

Self-reported HRQOL outcomes were collected using Qualtrics at T0, T1, T2, and T3. Participants received up to three reminders per survey. Measures were selected based on our theoretical framing that student mental health comprises both ill-being (e.g., anxiety, depression, and related distress domains) and well-being and functioning, including HRQOL and role/social functioning. The instrument set is listed in Table S1 and was previously used in a college student population described by Jayaraj et al. [9].

#### 2.7.2. App engagement and positive psychology intervention (PPI) activity exposure definitions

Overall app usage frequency, a proxy for engagement [20], was defined as any logged interaction on a given day: completion of a PPI activity, submission of a mood entry, creation of a forum post, or viewing a forum post. App usage frequency was calculated as the summed number of these events across the study period; partially completed PPI activities were not counted because timestamps of these occurrences were not available. PPI activity users were defined as participants who interacted with any PPI activity during the observation window, and non-users as those who did not.

#### 2.7.3. Fitbit^®^ wear-time

Fitbit^®^ Charge 5 devices generated accelerometer-based summary measures. Wear-time was estimated using the presence of heart rate data using the Fitbit^®^ API [21], expressed in hours (0–24 h) and percentages of the day [8,22]. For step count analysis, we calculated average daily steps with ≥6 h of wear-time between 8 AM and 8 PM. No wear-time cut-off was applied for sleep duration because average estimates were stable across cutoffs.

### 2.8. Qualitative interviews and thematic analysis

Participants were invited to complete an optional individual semi-structured exit interview within one month after the exit survey. Interviews were conducted April–June 2023 using a Health Insurance Portability and Accountability Act (HIPAA)-compliant U-M Zoom platform and lasted approximately 15 min [23]. Interviewers (ULS, JK, and MK) followed a semi-structured guide (Table S2) and used follow-up probes to elicit detail. Interviews were audio-recorded, professionally transcribed verbatim (Babbletype Inc., Philadelphia, PA, USA), and de-identified prior to analysis.

We analyzed transcripts using thematic analysis [24], consistent with our prior work [25,26]. Two or more team members reviewed and coded transcripts using NVivo Pro 13 (QSR International, Burlington, MA, USA). Analysis proceeded iteratively through transcript familiarization, initial code generation, theme development using both deductive and inductive approaches, theme review/refinement, and final theme definition and naming [24]. Coding consistency and theme coherence were reviewed by at least two team members, and final interpretive decisions were made collaboratively (J.K., M.K., U.L.S., and S.W.C.). The interview codebook is provided in Tables S3 and S4.

### 2.9. Mixed-methods integration

Using a convergent mixed-methods approach [27], quantitative and qualitative data were collected and analyzed separately and then merged to support integrated interpretation. Integration was conducted by aligning qualitative themes with quantitative engagement patterns and outcome trends through a joint display, with the goal of contextualizing engagement results and identifying implementation barriers and opportunities for improvement.

### 2.10. Exploratory analyses

Descriptive statistics were summarized as frequencies and percentages for categorical variables and means/SDs (or medians/IQRs where appropriate) for continuous variables. Fisher’s exact tests evaluated associations between categorical variables. The Kruskal–Wallis test assessed group differences across three or more levels. For pre–post comparisons of PROMIS^®^ scores, paired t-tests were used, with False Discovery Rate (FDR) adjustment via the Benjamini–Hochberg (BH) procedure [28]. PROMIS^®^ T-scores were calculated using the HealthMeasures scoring system [29].

To visualize patterns of PPI activity combinations, we used an UpSet plot (RStudio 4.4.3, Posit Software, PBC, Boston, MA, USA) [30]. To examine short-term mood trajectories around PPI activity engagement, we used a hinge plot comparing three days before versus three days after the activity index day.

For exploratory modeling, we aimed to reduce the dimensionality of correlated PROMIS^®^. Principal Component Analysis (PCA) was conducted, which yielded two orthogonal components (PC1 and PC2). The first two components (PC1 and PC2) explained 32.48% and 26.83% of the variance, respectively, accounting for a total of 59.31% of the variance in the data. A biplot was subsequently generated to visualize the projection of all PROMIS^®^ features onto a two-dimensional space. To enhance interpretability, a varimax rotation was applied to the components, resulting in rotated components (RC1 and RC2) that aligned with the x- and y-axes. Generalized Estimating Equations (GEEs) were employed to estimate differences in mood change slopes between PPI activity users and non-users (as defined below). Additionally, a t-test was used to assess differences in mood scores over time.

An exploratory longitudinal logistic mixed-effects model was fitted to investigate the association between daily app usage (binary: 0 or 1) and the rotated components (RC1 and RC2). An exploratory linear mixed-effects model was fitted to investigate the association between mood and physical activities. Since mood was recorded daily and PROMIS^®^ was only assessed at four specific points during the study, RC1 and RC2 (obtained from varimax) were treated as repeated measures over 30 time points, aligning it with the mood data from the preceding 30 days before each survey completion date. An exploratory linear mixed-effects model was used to assess whether there was a change in mood following each PPI activity, using a hinge model to allow for a continuous mood trajectory with a change in slope after the activity. These analyses were exploratory. Statistical tests used a 0.05 threshold and are interpreted as hypothesis-generating. The *p*-values are presented as exploratory signals in a small pilot and should not be interpreted as definitive evidence. All analyses were performed using statistical software R (version 4.5.1) and the LME4 package.

## 3. Results

### 3.1. Participant characteristics

Twenty-eight participants enrolled in this single-arm pilot study. Of these, 22 attended at least one coaching session and 15 completed an exit interview (these are overlapping subsets of the total enrolled cohort). The median age was 22 years (range, 18–36). Most participants were female (*n* = 18, 64%) and undergraduate students (*n* = 18, 64%). Using Fisher’s exact test, there were *no* statistically significant differences between the full cohort and the subsets who completed interviews or participated in coaching (Table 1).

### 3.2. Exploratory engagement with intervention components

#### 3.2.1. Roadmap 2.0 app engagement

**Figure 2.**
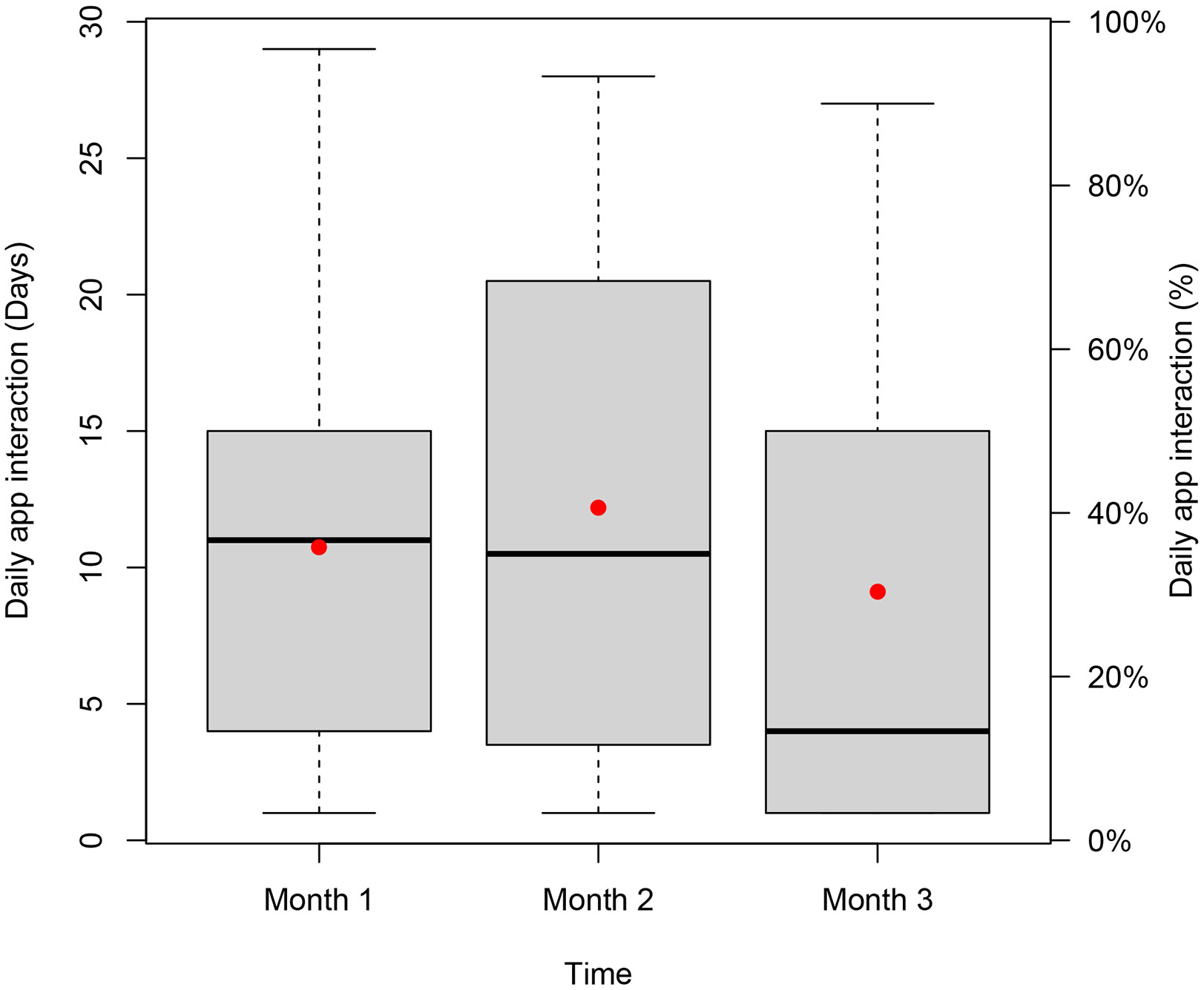
Daily app interaction over time. Daily app interaction rates are illustrated over the course of three months, based on a binary indicator of usage (1 = used app; 0 = did not use). A day was considered “app used” if the participant engaged in any of the following: completed a positive activity, entered a mood, created a post, or viewed a post. Median = black line; mean = red dot (days).

Daily app engagement was defined a priori as any of the following recorded actions: completing a positive activity, entering a mood score, creating a chat forum post, or viewing a chat forum post. Using this definition, participants engaged with the app on a median of 11 days at T1 and 11 days at T2 (means = 11 and 12 days, respectively). Engagement declined at T3 (median = 4 days, and mean = 9 days), with increased variability across participants (Figure 2).

#### 3.2.2. Wellness coaching participation

Participants could schedule coaching sessions at their discretion. Among those who attended coaching, participants completed a mean of two sessions during the study period (range: 1–5). Six participants did not attend any coaching sessions, most commonly citing scheduling conflicts.

### 3.3. Exploratory changes in self-reported outcomes over time

Across the 4-month duration of the study, we observed descriptive changes in PROMIS^®^ scores from baseline to exit. After multiple-comparison adjustment, global mental health (Mean [Standard Error (SE)]: 6.36 [2.4]; *p* adjusted [p*] = 0.049) and positive affect increased (Mean [SE]: 10.29 [3.52]; p* = 0.046), and depression (Mean [SE]: −9.01 [2.98]; p* = 0.043), anxiety (Mean [SE]: −9.12 [2.71]; p* = 0.04), fatigue (Mean [SE]: −8.91 [3.48] p* = 0.049), and anger (Mean [SE]: −9.03 [3.48] p* = 0.049) decreased. The other domains assessed were non-significant and are available in Table S5.

### 3.4. Exploratory construction of composite dimensions for modeling (Principal Component Analysis (PCA))

**Figure 3.**
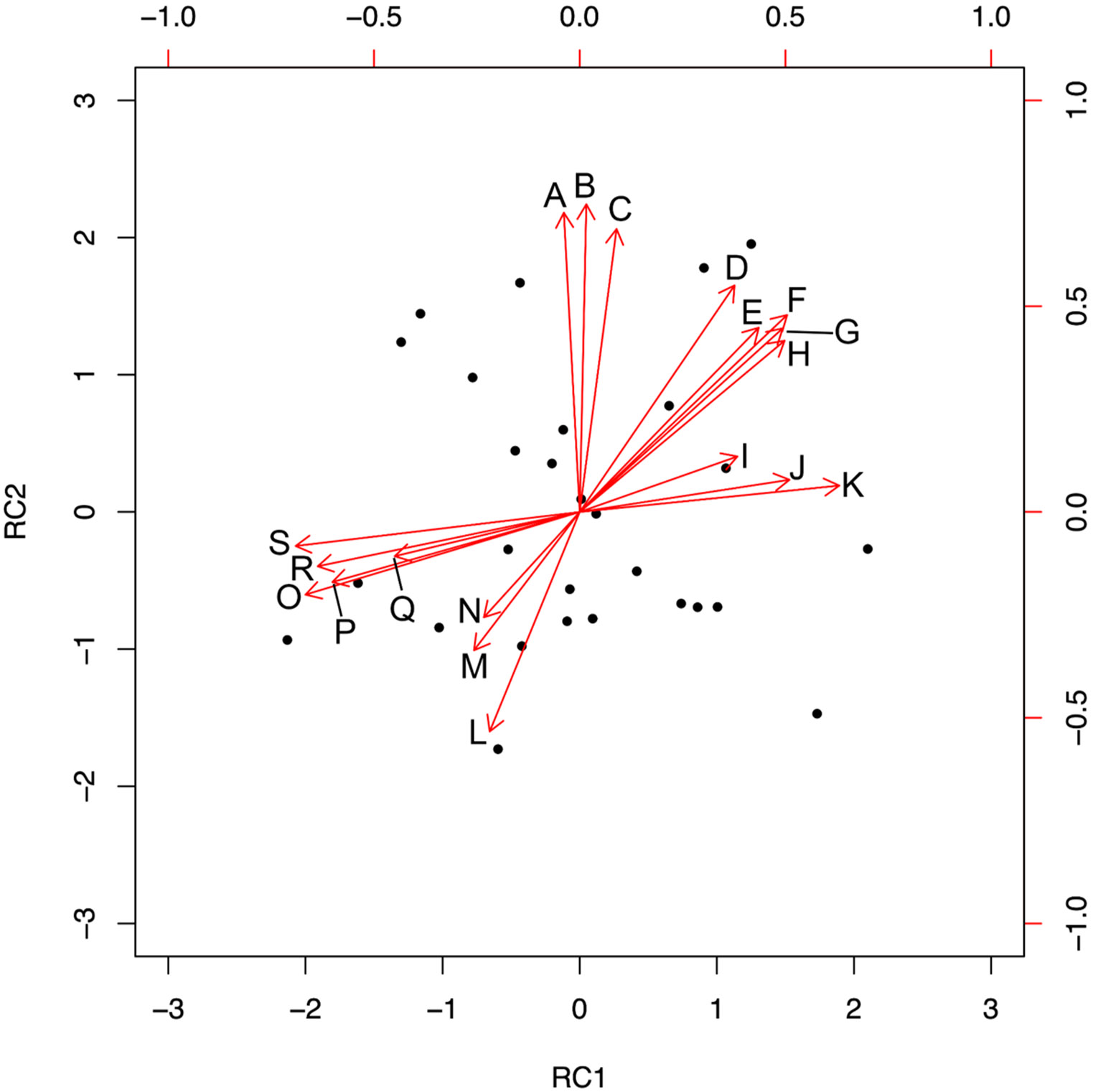
Biplot of PROMIS^®^ variables. In the biplot exploration of observed data, each vector represents a variable domain, and the orientation and length of the arrows indicate the strength and direction of their loadings on the two principal axes, RC1 and RC2, the first two varimax-rotated principal components. RC1 (x-axis) reflects a distress/burden dimension (higher loadings for depression, anxiety, fatigue, sleep-related impairment, and anger). RC2 (y-axis) reflects psychosocial resources/support (higher loadings for emotional support, informational support, companionship, and satisfaction with social roles and activities). The dots are the individual contributions to PROMIS. For example, if the dot is close to line O, that means this person contributes the most to this PROMIS. Variables pointing in the same direction tend to be positively related, while those in opposite directions tend to be negatively related. The variables are labeled as follows: (A) Informational Support; (B) Emotional Support; (C) Companionship; (D) Satisfaction with Social Roles and Activities; (E) Global Mental Health; (F) Meaning Purpose; (G) Positive Affect; (H) Global Physical Health; (I) Physical Function; (J) Ability to Participate in Social Roles and Activities; (K) Cognitive Function; (L) Social Isolation; (M) Pain Interference; (N) Sleep Disturbance; (O) Depression; (P) Sleep-Related Impairment; (Q) Anger; (R) Anxiety; (S) Fatigue.

To reduce the dimensionality of PROMIS^®^ domains for subsequent exploratory modeling, PCA was performed with varimax rotation and derived two rotated components (RC1 and RC2; Figure 3). RC1 captured a distress/burden dimension (higher loadings for depression, anxiety, fatigue, sleep-related impairment, and anger), whereas RC2 captured psychosocial resources and support (higher loadings for emotional support, informational support, companionship, and satisfaction with roles and activities). Global functioning measures (e.g., global mental health, global physical health, meaning and purpose) were loaded in the positive RC1 and RC2 quadrants, providing a suitable framework for subsequent regression modeling.

### 3.5. Exploratory Roadmap 2.0 app engagement and coaching participation

#### 3.5.1. Exploratory app engagement

In the exploratory mixed-effects logistic regression model (Table 2), engagement declined over time (log odds ratio −0.025, *p* < 0.001). A higher RC2 was associated with lower odds of app use (log odds ratio −0.533, *p* < 0.001). RC1 showed a marginal association with engagement (log odds ratio −0.193, *p* = 0.081). Sex was not significantly associated with app engagement.

#### 3.5.2. Exploratory coaching participation

In a parallel exploratory mixed-effects model of coaching participation (Table 3), no associations reached statistical significance. There were non-significant trends suggesting greater participation among female students (log odds ratio 1.034, *p* = 0.060) and participants with higher RC1 scores (log odds ratio 0.436, *p* = 0.080). RC2 showed a positive but non-significant association with coaching participation (log odds ratio 0.396, *p* = 0.123). Time was not significantly associated with coaching session attendance (log odds ratio −1.206, *p* = 0.076).

### 3.6. Exploratory engagement with PPI activities and mood

#### 3.6.1. Exploratory evaluation of which activities were used

**Figure 4.**
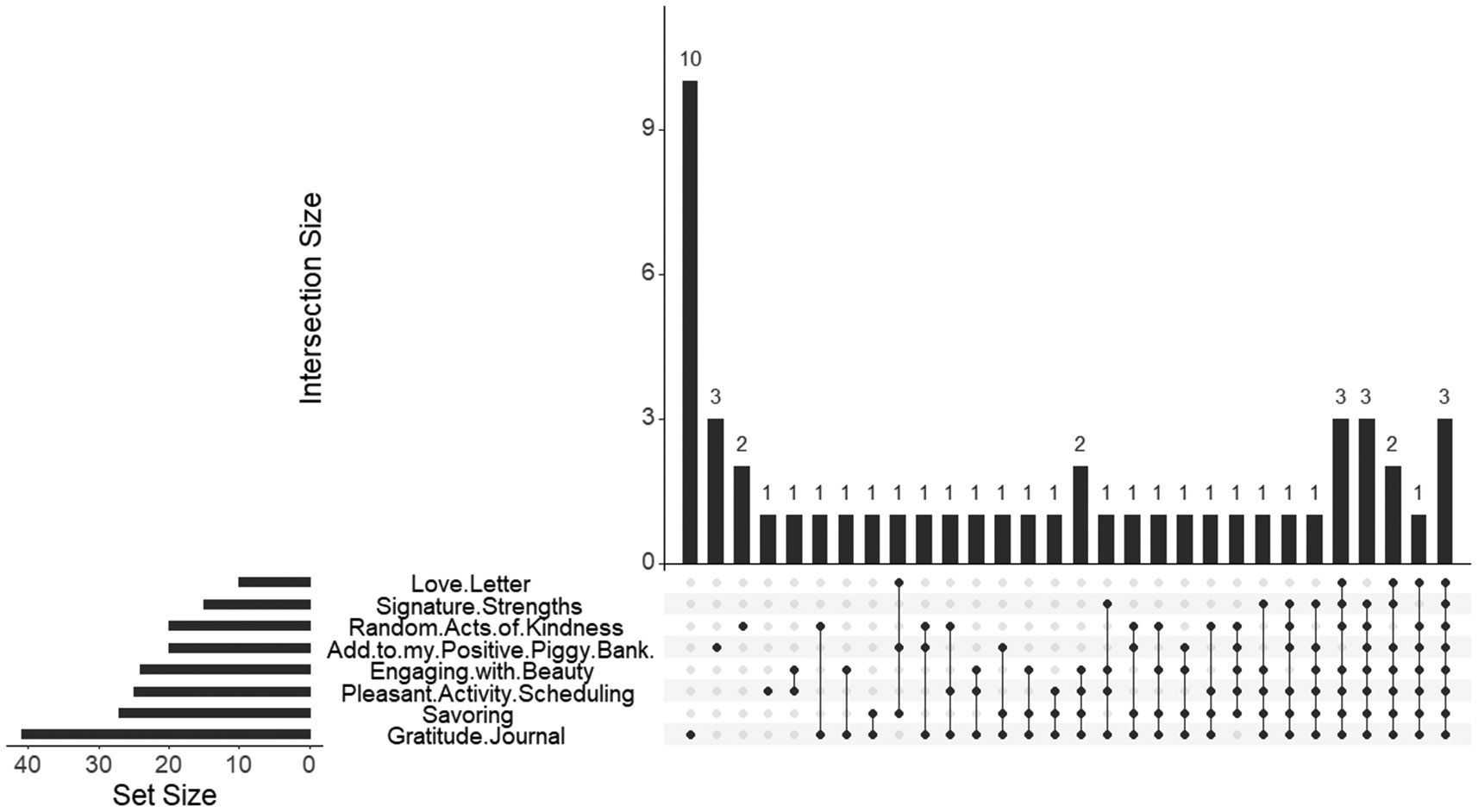
UpSet Plot for positive psychology intervention (PPI) activity engagement patterns. This UpSet plot visualizes the intersection of the positive activities engaged with by students using the app. The x-axis represents unique combinations of activities, while the y-axis indicates the number of users for each combination. The dots present in each column represent the use of the corresponding activity in the same row. Of note, single PPI activity usage is represented by the Set Size on the left: “Positive Piggy Bank” (12 unique users), “Savoring” (12 users), “Engaging with Beauty” (10 users), “Gratitude Journal” (9 unique users), and “Pleasant Activity Scheduling” (9 users) were the most used, while “Random Acts of Kindness” (3 users), “Signature Strengths” (3 users), and “Love Letter” (3 users) were the least used.

We next examined exploratory engagement specifically with the app’s PPI activities. Participants completed a range of activity combinations across the study period (Figure 4). The most commonly completed activities were the Positive Piggy Bank, Savoring, Engaging with Beauty, Gratitude Journaling, and Pleasant Activity Scheduling.

#### 3.6.2. Exploratory short-term mood changes around PPI activity use

**Figure 5.**
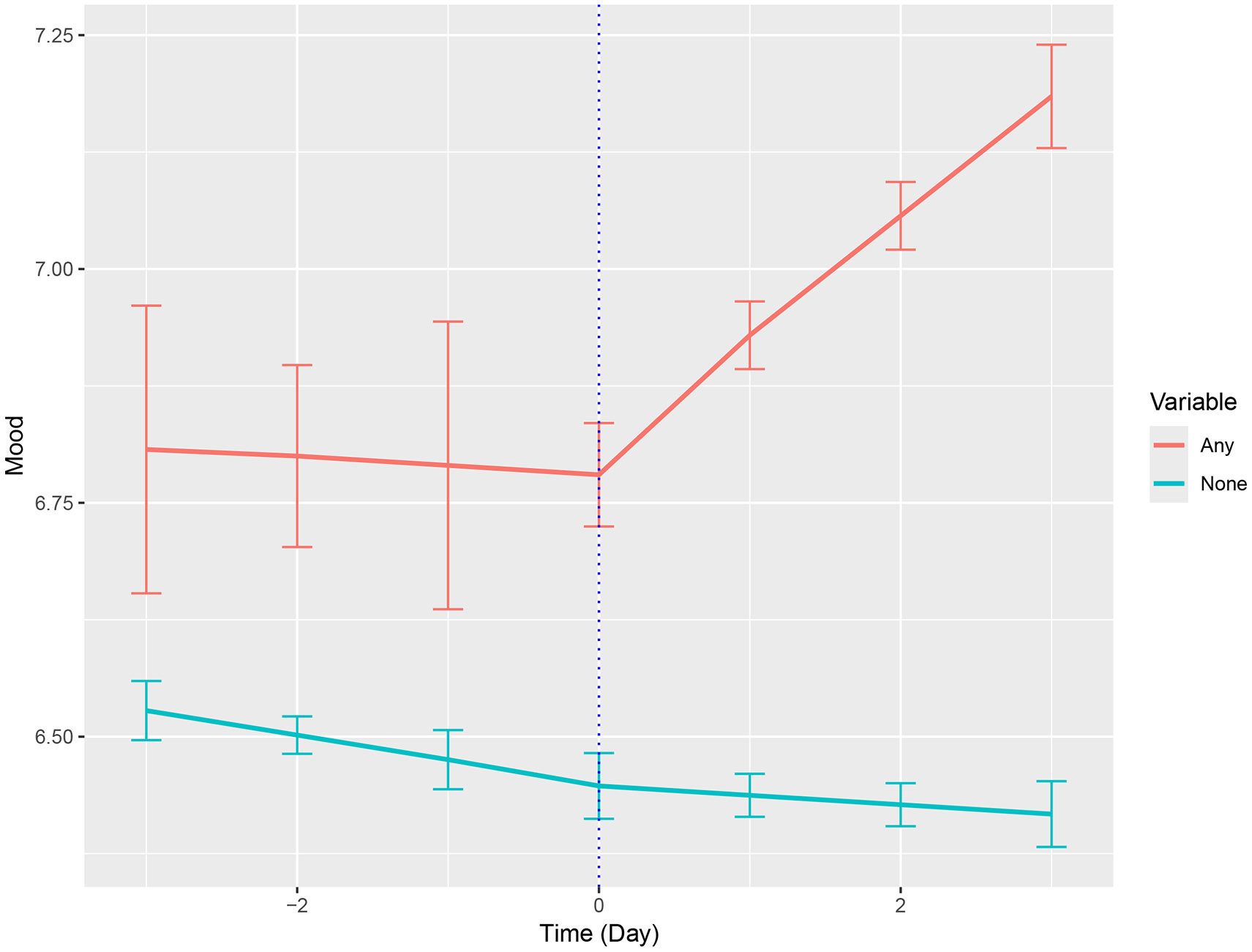
Hinge plot of mood scores before and after positive psychology intervention (PPI) activity engagement. Participants were grouped into two categories for this hinge plot: those who used any PPI activity (PPI activity users) and those who did not engage in any activities during the same window (non-users). The day they reported the activity is denoted here as Day 0 or the index day. x-axis: Time (day); y-axis: Mood Scores (1–10).

To explore temporal mood patterns around PPI activities, we constructed a hinge plot comparing mood trajectories from three days before to three days after any activity (Figure 5). Mood in the PPI activity users was relatively stable prior to the index day and trended upward in the three days following engagement, whereas mood in the non-users showed a gradual decline across the same window.

#### 3.6.3. Exploratory mood trajectories across the full study period

**Figure 6.**
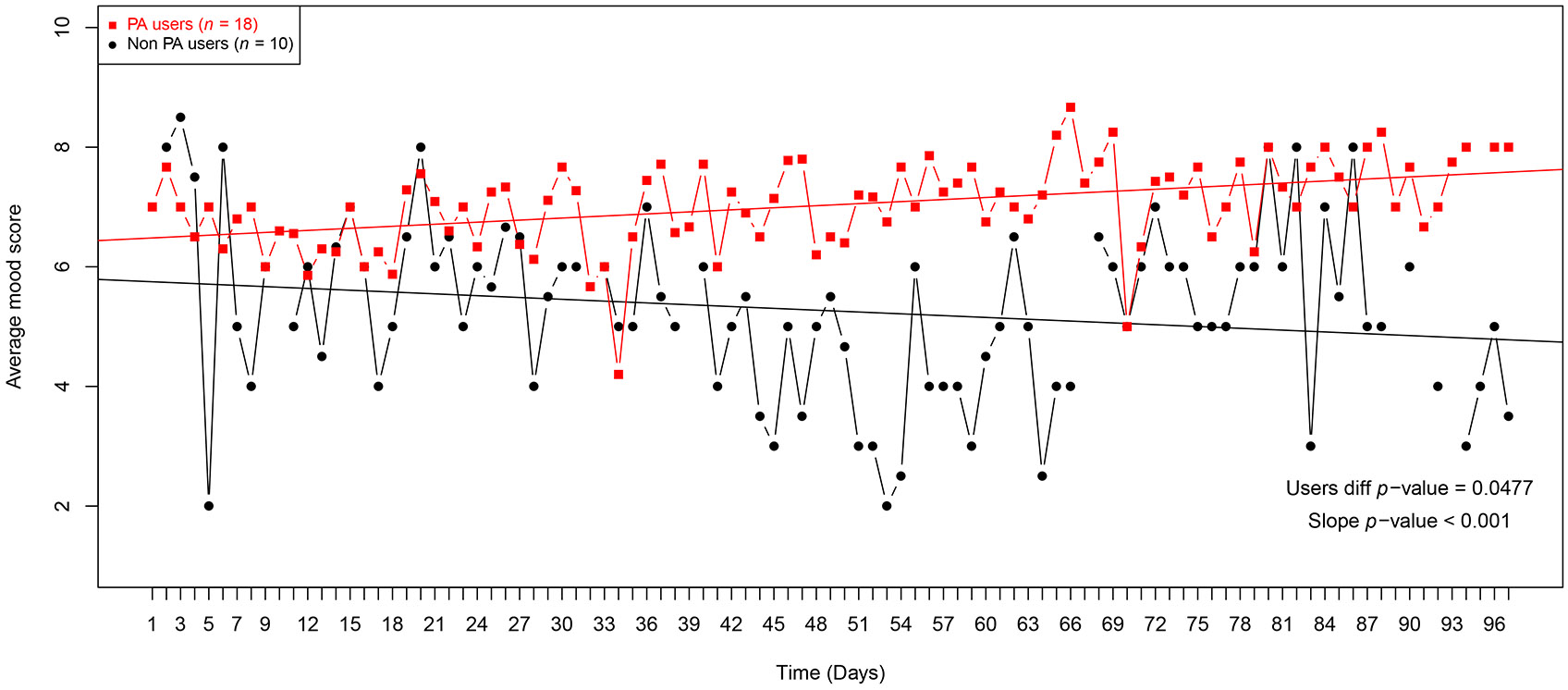
Exploratory change in mood scores in study participants over time. There are two groups represented in this figure. Positive psychology intervention (PPI) activity users represent students who engaged in positive activities (*n* = 18; red), and non-users represent students who did not engage in any positive activities (*n* = 10; black). PPI activity users had higher mood ratings than non-users, *p* = 0.048; the slopes also differed significantly, *p* < 0.001. PA: positive activities.

Over time, PPI activity users (*n* = 18) had higher average mood ratings than non-users (*n* = 10) (Figure 6). The difference between groups was statistically significant (*p* = 0.048) and the slopes of the trends differed significantly (*p* < 0.001).

There were no significant differences in the trajectories or slopes of step counts or sleep duration (in minutes) over the course of the study (Figures S1 and S2, respectively). However, students who engaged in PPI activities had a higher average step count than those who did not participate in any PPI activities (Figure S1).

### 3.7. Exploratory qualitative findings and integration with quantitative results

Exploratory interviews provided context for engagement patterns and outcome trends. There was convergence between objective engagement metrics (app timestamps and coaching sessions) and participant narratives, as shown in the joint table (Table S6). Participants described synergy between app-based reflection and wellness coaching, using the app to process feelings and arrive at coaching better prepared. This aligned with recorded use across intervention components and generally positive overall feedback. PROMIS^®^ findings and longitudinal mood data aligned with interview reports of perceived improvements in global mental health and reduced depression, anxiety, fatigue, and mood symptoms, with coaching frequently cited as supportive and integrative. App feature-level engagement mapped to qualitative accounts that the app supported habit formation and that the community/peer content increased motivation to use the app. Both quantitative and qualitative data highlighted implementation barriers and areas for improvement, engagement, and coaching participation were modest over time (i.e., average two coaching sessions), and users reported time constraints, difficulties scheduling via the portal, technical glitches, and a desire for more customizable/usable notifications and clearer, more user-friendly onboarding (Table S6).

## 4. Discussion

In this pilot study, we evaluated a blended intervention combining a digital PPI app (Roadmap 2.0) with optional wellness coaching among college students at a public university in the Midwest. The main findings suggest favorable descriptive trends over time and associations between engagement measures and outcomes. Interestingly, the exploratory results indicate that students who engaged more actively with the Roadmap 2.0 app’s suite of PPI activities had higher mood ratings and different mood trajectories compared to those who did not utilize these features. The exploratory upward mood trajectory among PPI activity users contrasted with the decline in slope in non-users. This observation is consistent with the literature suggesting that brief, app-delivered PPIs may be associated with improved emotional well-being [31]. Furthermore, the exploratory hinge plot analysis showed a post-activity increase in mood. This pattern was not observed among non-users. Notably, exploratory Fitbit^®^ data also indicated that PPI activity users had higher average step counts than non-users.

While both app use and participation in coaching sessions declined over time, a common pattern in digital mental health interventions [32], interviews described perceived app–coaching synergy. Participants described the app’s PPI activities as useful prompts for self-reflection and habit formation, and wellness coaching as a space for guided goal setting and processing personal challenges. The combined intervention was perceived as meaningful, consistent with prior research on the potential added value of blended digital and human support for mental health and well-being [33].

To further explore the underlying relationships among psychological and health-related variables, we conducted a biplot analysis that identified two key components: psychological distress and burden (RC1), and psychosocial support and available resources (RC2). Burden-related symptoms grouped in RC1, while resource-oriented measures aligned with RC2. Exploratory quantitative modeling revealed that higher RC1 scores were marginally associated with greater app engagement and session attendance, suggesting that students experiencing greater psychological burden were more likely to utilize both Roadmap 2.0 and wellness coaching. In contrast, greater perceived social resources and interpersonal connectedness (RC2) were associated with a reduced likelihood of app use. It is possible that students with stronger support networks may rely less on digital tools for mental health support. These findings suggest that engagement with the app and coaching may have been influenced by perceived psychological need, such as distress or social isolation, rather than demographic characteristics, as sex was not associated with use. Moreover, although no student characteristics significantly correlated with coaching attendance in this study, there was a trend toward greater participation among female students and those experiencing higher psychological burden. These insights could help identify which populations might be most likely to engage or find the approach acceptable and highlight areas for further exploration.

The integration of quantitative assessments with qualitative interview data enhanced our exploratory understanding of students’ lived experiences during the study period. Participants frequently noted that engaging with the app made them more intentional about self-care and provided a tool to manage stress, while wellness coaching offered accountability and personalized reflection. Nonetheless, several challenges were identified, including barriers to scheduling coaching sessions (six students did not attend any coaching sessions due to scheduling conflicts or being too busy), technical glitches with the app, and a lack of targeted notifications to prompt ongoing engagement. These factors, combined with academic time constraints, contributed to attrition in both app and coaching participation, consistent with the declining timestamps.

Previous studies have explored the use of mHealth apps [14,15,34] or various forms of wellness coaching individually in student populations [35,36]. The blended approach developed for this pilot study is a potentially meaningful advancement in how student wellness can be monitored and supported, particularly with the emergence of AI tools or chatbots. Notably, according to the American Psychological Association [37], students are seeking out mental health resources and accessing counseling centers at record rates. As counseling centers face increasingly high caseloads without corresponding increases in funding, there is a growing need for innovative solutions. This demand was evident in our study, as students readily enrolled and provided positive feedback about their experiences.

### 4.1. Strengths and limitations

A key strength of this study was the rigor applied in data collection and analysis, leveraging a convergent mixed-methods design to ensure a comprehensive understanding of outcomes. By integrating quantitative data, such as self-reported surveys and objectively measured Fitbit^®^ data, with qualitative interview insights, this study leveraged the strengths of both approaches. This multi-modal assessment allowed findings to be interpreted from multiple perspectives, ultimately enabling a richer exploration of participant experiences.

Nonetheless, several limitations should be considered when interpreting these exploratory findings. Firstly, all survey data, including daily mood ratings and PROMIS^®^ assessments, were self-reported, which may introduce response bias and affect data accuracy over time. App use or engagement with the PPI activities may have been underestimated, as tracking was limited to completed activities or submitted mood ratings. Some participants reported engaging with the app without formally completing or submitting their use, insights only captured in interviews. Additionally, the study’s single-arm design, small sample size, and conduct at a Midwestern public institution limit the generalizability of the results. Selection bias is also possible, as participants self-selected for wellness coaching and may represent a more help-seeking or self-motivated subpopulation. The absence of a control group precludes causal inference, and the relatively short follow-up may not capture sustained or long-term effects. The exploratory analyses included multiple comparisons and potential overfitting in mixed-effects models given the small sample size and repeated measures. Finally, as the intervention leveraged a university-specific resource, Wolverine Wellness, a free resource available to these study participants, access and outcomes may differ at less resourced institutions lacking similar support services.

Despite these limitations, our findings provide preliminary data supporting the potential feasibility and acceptability of combining digital PPIs with human coaching support for college students. The program was well received, with nearly all participants expressing satisfaction with their involvement and reporting meaningful positive changes. Several key design considerations for future interventions emerged: ensuring technical stability, streamlining access to coaching, and developing tailored engagement strategies may further enhance program effectiveness and retention.

## 5. Conclusions

The pilot study herein supports the feasibility of a blended approach and suggests the potential for favorable outcome trends in college student mental health and well-being. Our findings suggest that integrating the two approaches may support emotional reflection and is associated with favorable trends in self-reported outcomes. While participants responded positively to the blended intervention, this study’s small, self-selected sample, single-site design, and reliance on self-reported measures limit the generalizability of these results. Future, larger-scale randomized controlled trials are necessary to confirm these findings, clarify mechanisms of action, and determine best practices for sustained engagement and maximal benefit. A subsequent multi-site, randomized controlled trial has been registered (NCT06804213) to evaluate efficacy and isolate component contributions. This study expands the current work by including multiple sites, a more diverse participant pool, and a rigorous comparison between the combined app plus coaching approach and traditional human coaching alone. This trial is designed to provide further insight into the scalability and efficacy of integrating mHealth tools and personalized support within university wellness programs to meet the escalating mental health needs of college students.

## Supplementary Material

Supplemental Materials

Supplemental Figure 1

Supplemental Figure 2

Supplemental Figure 3

The supplementary materials are available at https://doi.org/10.20935/xxx. Reference [38] is cited in the Supplementary materials.

## Figures and Tables

**Table 1.** Participant characteristics. N/A: not applicable.

	In study (*N* = 28)	Interviewed (*n* = 15)	Attended coaching (*n* = 22)	*p*-value
**Median age (range)**	22 (18–36)	22 (19–36)	22 (18–36)	0.568
**School year**				0.766
Undergraduate	18 (64.3%)	8 (53.3%)	13 (59.1%)	
Graduate	10 (35.7%)	7 (46.7%)	9 (40.9%)	
**Sex**				0.809
Woman	18 (64.3%)	9 (60.0%)	12 (54.5%)	
Man	10 (35.7%)	6 (40.0%)	10 (45.5%)	
**Race**				0.653
White	9 (32.1%)	5 (33.3%)	8 (36.4%)	
Black or African American	5 (17.9%)	2 (13.3%)	4 (18.2%)	
Asian	11 (39.3%)	7 (46.7%)	8 (36.4%)	
Biracial	2 (7.1%)	1 (6.7%)	1 (4.5%)	
Not reported	1 (3.6%)	N/A	1 (4.5%)	
**Ethnicity**				0.999
Hispanic	3 (10.7%)	2 (13.3%)	2 (9.1%)	
Not Hispanic	24 (85.7%)	13 (86.7%)	20 (90.9%)	
Not reported	1 (3.6%)	N/A	N/A	
**Domestic/international**				0.771
Domestic	19 (67.9%)	9 (60.0%)	12 (54.5%)	
International	8 (28.6%)	6 (40.0%)	5 (22.7%)	
Not reported	1 (3.6%)	N/A	5 (22.7%)	
**Generation**				0.902
First generation	4 (14.3%)	2 (13.3%)	5 (22.7%)	
Continuing generation	11 (39.3%)	6 (40.0%)	9 (40.9%)	
Not reported	13 (46.4%)	7 (46.7%)	8 (36.4%)	

**Table 2.** An exploratory mixed-effects logistic regression model to examine associations with daily app use.

Variable	Estimate	*p*-value
Day	−0.0253	<0.001***
**Sex**		
Male	Ref	Ref
Female	0.2883	0.6924
RC1	−0.1935	0.0807
RC2	−0.5326	<0.001***

“Ref” represents the reference category for the corresponding variable. Significance is indicated with stars:

*** for *p* < 0.001. RC1 reflects a distress/burden dimension (higher loadings for depression, anxiety, fatigue, sleep-related impairment, and anger). RC2 reflects psychosocial resources/support (higher loadings for emotional support, informational support, companionship, and satisfaction with social roles and activities).

**Table 3.** An exploratory mixed-effects logistic regression model to examine associations with wellness coaching.

Variable	Estimate	*p*-value
Day	−1.2064	0.0755
**Sex**		
Male	Ref	Ref
Female	1.0337	0.0603
RC1	0.4364	0.0799
RC2	0.3957	0.1231

“Ref” represents the reference category for the corresponding variable. RC1 reflects a distress/burden dimension (higher loadings for depression, anxiety, fatigue, sleep-related impairment, and anger). RC2 reflects psychosocial resources/support (higher loadings for emotional support, informational support, companionship, and satisfaction with social roles and activities).

## Data Availability

The data supporting the findings of this publication can be made available upon request.

## References

[1] American College Health Association. American College Health Association-National College Health Assessment III: reference group executive summary Spring 2025. Silver Spring, MD, USA: American College Health Association; 2025.

[2] Healthy Minds Network. Healthy minds study among colleges and universities, year 2023–2024. Healthy Minds Network, University of Michigan, University of California Los Angeles, Boston University, and Wayne State University; 2025. Available from: https://healthymindsnetwork.org/research/data-for-researchers

[3] National Academies of Sciences, Engineering, and Medicine, Health and Medicine Division, Policy and Global Affairs, Board on Health Sciences Policy, Board on Higher Education and Workforce, Committee on Mental Health, Substance Use, and Wellbeing. Mental health, substance use, and wellbeing in higher education: supporting the whole student. Washington, DC, USA: National Academies of Sciences, Engineering, and Medicine; 2021. doi: 10.17226/26015

[4] Seligman MEP. Flourish: visionary new understanding happiness well-being. New York, NY, USA: Free Press; 2011.

[5] Westerhof GJ, Keyes CLM. Mental illness and mental health: the two continua model across the lifespan. J Adult Dev. 2010; 17 (2): 110–9. doi: 10.1007/s10804-009-9082-y 20502508 PMC2866965

[6] Moè A. Does the weekly practice of recalling and elaborating episodes raise well-being in university students? J Happiness Stud. 2022; 23 (1): 3389–406. doi: 10.1007/s10902-022-00547-w 35818379 PMC9258475

[7] Kern ML, Waters LE, Adler A, White MA. A multidimensional approach to measuring well-being in students: application of the PERMA framework. J Posit Psychol. 2015; 10 (3): 262–71. doi: 10.1080/17439760.2014.936962 25745508 PMC4337659

[8] Gilley KN, Baroudi L, Yu M, Gainsburg I, Reddy N, Bradley C, Risk factors for COVID-19 in college students identified by physical, mental, and social health reported during the fall 2020 semester: observational study using the Roadmap app and Fitbit wearable sensors. JMIR Ment Health. 2022; 9 (2): e34645. doi: 10.2196/34645 34992051 PMC8834863

[9] Jayaraj G, Cao X, Horwitz A, Rozwadowski M, Shea S, Hanauer SN, Trends in mental health outcomes of college students amid the pandemic (Roadmap mHealth app): longitudinal observational study. J Med Internet Res. 2025; 27: e67627. doi: 10.2196/67627 39787592 PMC11757984

[10] Sin NL, Lyubomirsky S. Enhancing well-being and alleviating depressive symptoms with positive psychology interventions: a practice-friendly meta-analysis. J Clin Psychol. 2009; 65(5): 467–87. doi: 10.1002/jclp.20593 19301241

[11] Fält-Weckman S, Fagerlund Å, Londen M, Lagerström M. Using evidence-based applied positive psychology to promote student well-being. Front Psychol. 2024; 15: 1415519. doi: 10.3389/fpsyg.2024.1415519 38988385 PMC11234798

[12] Braunwalder C, Müller R, Glisic M, Fekete C. Are positive psychology interventions efficacious in chronic pain treatment? A systematic review and meta-analysis of randomized controlled trials. Pain Med. 2022; 23 (1): 122–36. doi: 10.1093/pm/pnab247 34347095

[13] Bolier L, Majo C, Smit F, Westerhof GJ, Haverman M, Walburg JA, Cost-effectiveness of online positive psychology: randomized controlled trial. J Posit Psychol. 2014; 9 (5): 460–71. doi: 10.1080/17439760.2014.910829

[14] Choudhury A, Kuehn A, Shamszare H, Shahsavar Y. Analysis of mobile app-based mental health solutions for college students: a rapid review. Healthcare. 2023; 11 (2): 272. doi: 10.3390/healthcare11020272 36673640 PMC9859497

[15] Oliveira C, Pereira A, Vagos P, Nóbrega C, Gonçalves J, Afonso B. Effectiveness of mobile app-based psychological interventions for college students: a systematic review of the literature. Front Psychol. 2021; 12: 647606. doi: 10.3389/fpsyg.2021.647606 34045994 PMC8144454

[16] Mobile fact sheet. Pew Research Center; 2024. Available from: http://www.pewresearch.org/internet/fact-sheet/mobile/

[17] Lattie EG, Adkins EC, Winquist N, Stiles-Shields C, Wafford QE, Graham AK. Digital mental health interventions for depression, anxiety, and enhancement of psychological well-being among college students: systematic review. J Med Internet Res. 2019; 21 (7): e12869. doi: 10.2196/12869 31333198 PMC6681642

[18] University of Michigan Wolverine Wellness. Welcome to Wolverine Wellness. Wolverine Wellness—University of Michigan. Available from: https://wolverinewellness.umich.edu/

[19] Ju H, Kang E, Kim Y, Ko H, Cho B. The effectiveness of a mobile health care app and human coaching program in primary care clinics: pilot multicenter real-world study. JMIR mHealth uHealth. 2022; 10 (5): e34531. doi: 10.2196/34531 35522461 PMC9123543

[20] Szinay D, Jones A, Chadborn T, Brown J, Naughton F. Influences on the uptake of and engagement with health and well-being smartphone apps: systematic review. J Med Internet Res. 2020; 22 (5): e17572. doi: 10.2196/17572 32348255 PMC7293059

[21] Fitbit development: Web API 2023. Available from: https://dev.fitbit.com/build/reference/web-api/

[22] Faust L, Purta R, Hachen D, Striegel A, Poellabauer C, Lizardo O, Exploring compliance: observations from a large scale Fitbit study. In: Proceedings of the 2nd International Workshop on Social Sensing; 21 April 2017; Pittsburgh, PA, USA. New York, NY, USA: ACM; 2017. p. 55–60. doi: 10.1145/3055601.3055608.

[23] University of Michigan Information and Technology Services. Zoom at U-M. Available from: https://its.umich.edu/communication/videoconferencing/zoom

[24] Braun V, Clarke V. Using thematic analysis in psychology. Qual Res Psychol. 2006; 3 (2): 77–101. doi: 10.1191/1478088706qp063oa

[25] Smeallie E, Rosenthal L, Johnson A, Roslin C, Hassett AL, Choi SW. Enhancing resilience in family caregivers using an mHealth app. Appl Clin Inform. 2022; 13 (5): 1194–206. doi: 10.1055/a-1967-8721 36283418 PMC9771688

[26] Caparso C, Ozkan G, Kluge M, Salim H, Khaghany A, Blok A, Mobile technology to monitor and support health and well-being: qualitative study of perspectives and design suggestions from patients undergoing hematopoietic cell transplantation. JMIR Form Res. 2023; 7: e49806. doi: 10.2196/49806 37651172 PMC10502589

[27] Creswell JW. A concise introduction to mixed methods research. 2nd ed. Thousand Oaks, CA, USA: SAGE Publications; 2021.

[28] Benjamini Y, Hochberg Y. Controlling the false discovery rate: a practical and powerful approach to multiple testing. J R Stat Soc Series B Stat Methodol. 1995; 57 (1): 289–300. doi: 10.1111/j.2517-6161.1995.tb02031.x

[29] HealthMeasures. Available from: https://www.healthmeasures.net/

[30] Lex A, Gehlenborg N, Strobelt H, Vuillemot R, Pfister H. UpSet: visualization of intersecting sets. IEEE Trans Vis Comput Graph. 2014; 20(12): 1983–92. doi: 10.1109/TVCG.2014.2346248 26356912 PMC4720993

[31] Saboor S, Medina A, Marciano L. Application of positive psychology in digital interventions for children, adolescents, and young adults: systematic review and meta-analysis of controlled trials. JMIR Ment Health. 2024; 11: e56045. doi: 10.2196/56045 39141906 PMC11358669

[32] Smith KA, Ward T, Lambe S, Ostinelli EG, Blease C, Gant T, Engagement and attrition in digital mental health: current challenges and potential solutions. NPJ Digit Med. 2025; 8 (1): 398. doi: 10.1038/s41746-025-01778-w 40604240 PMC12223045

[33] Camacho E, Chang SM, Currey D, Torous J. The impact of guided versus supportive coaching on mental health app engagement and clinical outcomes. Health Inform J. 2023; 29 (4): 14604582231215872. doi: 10.1177/14604582231215872 38112116

[34] Nagar R, Quirk HD, Anderson PL. User experiences of college students using mental health applications to improve self-care: implications for improving engagement. Internet Interv. 2023; 34: 100676. doi: 10.1016/j.invent.2023.100676 37867616 PMC10587513

[35] Bleck J, DeBate R, Garcia J, Gatto A. A pilot evaluation of a university health and wellness coaching program for college students. Health Educ Behav. 2023; 50 (5): 613–21. doi: 10.1177/10901981221131267 36314384

[36] Yan Z, Peacock J, Cohen JFW, Kurdziel L, Benes S, Oh S, An 8-week peer health coaching intervention among college students: a pilot randomized study. Nutrients. 2023; 15 (5): 1284. doi: 10.3390/nu15051284 36904282 PMC10005245

[37] Abrams Z. Student mental health is in crisis. Campuses are rethinking their approach. Monit Psychol. 2022; 53 (7): 60. Available from: https://www.apa.org/monitor/2022/10/mental-health-campus-care

[38] Rozwadowski M, Dittakavi M, Mazzoli A, Hassett AL, Braun T, Barton DL, Promoting health and well-being through mobile health technology (Roadmap 2.0) in family caregivers and patients undergoing hematopoietic stem cell transplantation: protocol for the development of a mobile randomized controlled trial. JMIR Res Protoc. 2020; 9 (9): e19288. doi: 10.2196/19288 32945777 PMC7532463

