# Supplementary material for: Blended mobile health and wellness coaching enhances student engagement in mental health care
Source: Acad Ment Health Well Being. Author manuscript; Available in PMC 2026 Jun 26. (PMC13298186; doi:10.20935/mhealthwellb8298)
Supplement: Supplemental Materials [file NIHMS2181429-supplement-Supplemental_Materials.docx]

Supplementary Materials

Figure S1. Change in step counts in study participants over time. There are two groups represented in this figure. “PA” users represent students who engaged in positive psychology intervention (PPI) activities (*n* = 18; red) and “Non PA” users represent students who did not engage in any PPI activities (*n* = 10; black). There was no significant difference in the slope of sleep duration (in minutes) within groups, but participants who consistently engaged in PPI activities (PA) had a higher average step count than those who did not participate in any PPI activities (Non PA).

Figure S2. Change in Sleep Duration in Study Participants Over Time. There are two groups represented in this figure. “PA” users represent students who engaged in positive psychology intervention (PPI) activities (*n* = 18; red) and “Non PA” users represent students who did not engage in any PPI activities (*n* = 10; black). There were no significant differences between groups or change in slope within groups.


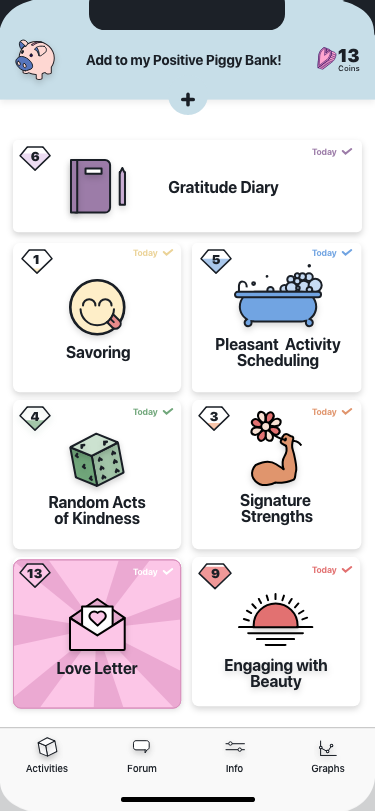


Figure S3. Roadmap 2.0 App Positive Psychology Intervention (PPI) Activity Screen. This figure is a sample of participant’s view of all the PPI activities available when logging into the Roadmap 2.0 application. The seven activities depicted include: Positive Piggy Bank, Gratitude Journal, Savoring, Pleasant Activity Scheduling, Random Acts of Kindness, Signature Strengths, Love Letter, and Engaging with Beauty [1]. The instructions given to participants for each activity is listed below along with examples of potential responses.

Sample text with instructions provided to the caregiver:

1. Positive Piggy Bank:

As human beings, we tend to focus on negative things, people and events. This focus on the negative can undermine our happiness. Keeping a Positive Piggy Bank can help us focus on all the good things in our world, too.

Step 1: When you observe something that makes you happy, take a moment as savor it. Think about what makes this so special to you.

Step 2: Make a note to capture this thing or moment with enough detail that you can immediately recall what happened later.

Step 3: Now, tap the coin and it will drop into your positive piggy bank.

Step 4: You can make as many of these happy memory “deposits” as you like. The best part is that when you need a little pick-me-up, you may “break” open your piggy bank and read all of these happy notes.

Examples:

Example 1: My best friend called to cheer me up. It worked!

Example 2: Peach pie with homemade whipped cream, yummy.

Example 3: My husband paid me the kindest compliment today.

Example 4: I love the feel of grass under my feet.

1. Gratitude Journal:

Feeling grateful is a powerful way to ward off depression and inspire feelings of optimism. It is perhaps the easiest positive emotion to tap in to when things are difficult. For that reason, we encourage you to keep a gratitude diary. You can do that right here! This is how to go about it:

Step 1: Every day, note at least 2 things for which you are grateful. It can be anything – your friends and family, your pets, feeling the sunshine on your face, happy that a friend phoned, receiving a present, being able to take a walk, chocolate cupcakes … anything. Evenings, right before you go to sleep, usually works best.

Step 2: Make a commitment to yourself that you will note at least 2 things every day, but here is a twist - the things you list MUST be DIFFERENT. Try never to repeat anything.

Step 3: Smile as you write these things down. This will help you to feel even more grateful.

There are examples within the instructions above, but here are some if more are needed:

Example of bigger things: My family

My health

Having enough food to eat

My dear friend, Terry

Example of smaller things: Children laughing

The beach

Peanut butter

Puppies!

1. Savoring:

Savoring involves recognizing special moments and taking efforts to make them last and be more memorable. You can savor food, experiences, moments with loved ones, anything that brings you pleasure.

Step 1: Consider a typical weekday. Review your morning routine, your daily activities, and your evening rituals, and consider how much time you spend noticing and enjoying the pleasures of the day, both small and large.

Step 2: Every day for the next week, be sure to savor at least two experiences (for example, your morning coffee, or the sun on your face as you walk to your car). Spend at least 2-3 minutes savoring each experience.

Step 3: Log these savoring experiences here so you can revisit them later.

Here are some more examples of things you could savor:

Sunsets

Fresh baked cookies

Time with your best friend

Visiting a new place

Playing your favorite game

A walk on a lovely day

1. Pleasant Activity Scheduling:

Providing care for loved ones can be incredibly time consuming. You might have already noticed that you have stopped doing many of the fun things you used to do. Yet, these pleasant activities are incredibly important and can help you better cope with stress. By scheduling and taking part in pleasant activities, you may find that you feel happier and have more energy.

Step 1: Identify activities that you find to be pleasant. These activities do not have to be expensive or time consuming – they just need to be things you enjoy. Activities could include taking a walk in the park, listening to music, working on your hobby, seeing a movie with a friend or reading a great book.

Step 2: Set aside time in the next week to do at least two of these activities. Put them on your calendar like an appointment and treat them with the same importance as you would a doctor’s appointment.

Step 3: Log what you did for your pleasant activity. Have fun, it’s good for you!

1. Random Acts of Kindness:

Although we do kind things daily, we often do not set out to intentionally do something nice for somebody else. Kindness is something always available for us to both give and receive.

Step 1: For this activity, one day this week, do five kind acts all in one day. Take a little time to plan what you are going to do. For the first ***four*** acts, do these for other people. These people can be complete strangers or friends and family members. These can be small acts of kindness such as holding a door open, sharing a genuine compliment or giving somebody a hug.

Step 2: You must also do ***one*** kind thing for you. People who take care of others tend to put them first and forget to be kind to themselves. It's important to take care of yourself, too! Perhaps, you could take a long bubble bath, go for a walk in the park, enjoy a Popsicle or sleep an extra 20 minutes.

Step 3: Smile as you do these kind acts. You are putting good into the world!

Here are some more ideas about kind acts you could do:

Leave inspiring message sticky notes to be found.

Give a friend a small gift for no particular reason.

Call a family member out of the blue and tell them how much they mean to you.

1. Signature Strengths:

Character strengths are connected with resilience and buffer people from vulnerabilities that can lead to depression and anxiety. Your unique set of character strengths make you, you. Using these strengths more regularly and in different ways can help you lead a more successful and rewarding life.

Step 1: Based on the Brief Strengths Test, note your top seven strengths.

Step 2: Every day for the next week, use one of these strengths in a way that you have not used it before.

Step 3: Each night, note how you used one of your strengths that day, including what strength you used, how you felt before, during, and after the activity, and whether you plan to repeat it in the future.

Examples:

If “creativity” is one of your strengths and you usually use creativity for arts and crafts, try using creativity to solve a difficult problem at work.

If “zest” is a strength of yours and you focus this on your hobby of cooking, try using zest to improve the quality of your friendships.

If “hope” is a strength of your and you tend to use hope to strengthen your faith, perhaps you could use hope in your exercise regime to envision the positive strides you can make.

1. Love Letter:

Finding ways to express warmth, care, deep positive regard, and authentic appreciation to those we love is important to us (the giver) to express, and for the receiver to hear and experience.

Step 1: Think about the love you have for the person for whom you are providing care.

Step 2: Write a brief love letter to this person. In the letter, tell your loved one about your love for him or her, offering your thoughts, feelings and specific examples. Also, consider linking your love to something that happened today or recently.

Step 3: Share your letter with the person you care for.

1. Engaging with Beauty:

Beauty in nature can inspire the emotion of ‘awe,’ beauty in art and skill can inspire admiration, and the witnessing of beauty in positive acts of human behavior can inspire more positive acts echoing like a ripple in a pond.

Step 1: Create a Beauty Log where you will add your observations about three different types of beauty: beauty in nature, beauty that is man-made (e.g., art, music, dance, architecture) or beautiful human behavior (e.g., kind acts, brave acts).

Step 2: Look for beauty as you go through the day. When you observe something that is beautiful, add it to your log in text or photo form.

1. Forum for Caregivers to post positive comments based on Positive Activity exercises that are shared with other Caregiver participants:
   1. Gratitude Room
   2. Acts of Kindness Room
   3. Observed Beauty Room
   4. Strengths Room
   5. Love Letter Room
2. Mood, Sleep, and Steps
   1. Patients and caregivers will be provided graphs of daily mood, sleep, and steps data (sample data plot shown on next page), depending on whether the participants respond to the daily mood questionnaire – scale between 1-10, anchored to “worst possible” and “best possible,” respectively – and wear the Fitbit® Charge.

**Table S1.** PROMIS® health-related quality of life (HRQOL) assessments by participants at baseline (T0), Month 1 (T1), Month 2 (T2), and Exit (End-of-Study; T3). SD: standard deviation, *T* score*:* standardized score.

| **Age 18+ years** | | |
| --- | --- | --- |
| **Measure** | **Description** | **Scoring** |
| PROMIS®  Global Health  (Mental and Physical) | 10-item Short Form represents Global Physical Health (overall physical health, physical function, pain, and fatigue) and Global Mental Health (quality of life, mental health, satisfaction with social activities and emotional problems). | *T* score (mean = 50; SD = 10); Two summary scores: Physical Health, Mental Health, higher scores indicate better health. |
| PROMIS®  Physical Function | 4-item Short Form assesses physical function (e.g., errands/chores, up/downstairs, walk). | *T* score (mean = 50; SD = 10); higher scores indicate better physical mobility. |
| PROMIS®  Companionship | 4-item Short Form assesses perceived availability of someone with whom to share enjoyable social activities. | *T* score with (mean = 50; SD = 10); higher scores indicate better companionship to share activities. |
| Neuro-QoL  (PROMIS® affiliate)  Positive Affect and Well-Being | 7-item Short Form assesses aspects of a person’s life that relate to a sense of well-being, life satisfaction or an overall sense of purpose and meaning. | *T* score (mean = 50; SD = 10); higher scores indicate better positive affect and well-being. |
| PROMIS®  Cognitive Function | 4-item Short Form assesses mental acuity, concentration, verbal and nonverbal memory, verbal fluency, and perceived changes in these cognitive functions. The extent to which cognitive impairments interfere with daily functioning, whether other people observe cognitive impairments, and the impact of cognitive dysfunction on quality of life are also assessed. | *T* score with (mean = 50; SD=10); higher scores indicate better cognitive functioning. |
| PROMIS®  Ability to Participate in Social Roles and Activities | 4-item Short Form assesses the perceived ability to perform one’s usual social roles and activities. | *T* score with (mean = 50; SD = 10); higher scores indicate better ability to participate in social roles and activities. |
| PROMIS®  Satisfaction with Participation in Social Activities | 4-item Short Form assesses the perceived contentment with leisure interests and relationships with friends. | *T* score with (mean = 50; SD = 10); higher scores indicate better satisfaction with participation in social activities. |
| PROMIS®  Emotional Support | 4-item Short Form assesses the availability of others with whom to talk with and feel appreciated by others. | *T* score (mean = 50; SD = 10); higher scores indicate better emotional support. |
| PROMIS®  Informational Support | 4-item Short Form assesses perceived availability of helpful information or advice. The item bank does not use a time frame (e.g., over the past seven days). | *T* score (mean = 50; SD = 10); higher scores indicate better informational support. |
| PROMIS®  Meaning and Purpose | 4-item Short Form assesses one’s sense of life having purpose and that there are good reasons for living. | *T* score (mean = 50; SD = 10); higher scores indicate hopefulness, optimism, goal-directedness, and feels that one’s life is worthy. |
| PROMIS®  Social Isolation | 4-item Short Form assesses perceptions of being avoided, excluded, detached, disconnected from, or unknown by others. The item bank does not use a time frame (e.g., over the past seven days) when assessing social isolation. | *T* score (mean = 50; SD = 10); higher scores indicate worse social isolation. |
| PROMIS®  Sleep Disturbance | 4-item Short Form assesses perceptions of sleep quality, sleep depth, and restoration associated with sleep. | *T* score (mean = 50; SD = 10); higher scores indicate worse sleep disturbance. |
| PROMIS®  Sleep-Related Impairment | 4-item Short Form assesses perceptions of alertness, sleepiness, and tiredness during usual waking hours, and the perceived functional impairments during wakefulness associated with sleep problems or impaired alertness. | *T* score (mean = 50; SD = 10); higher scores indicate worse sleep-related impairment |
| PROMIS®  Depression | 4-item Short Form that assesses self-reported negative mood (sadness, guilt), views of self (self-criticism, worthlessness), and social cognition (loneliness, interpersonal alienation). | *T* score (mean = 50; SD = 10); higher scores indicate worse depression. |
| PROMIS®  Anxiety | 4-item Short Form that assesses fear, anxious misery, hyperarousal, and somatic symptoms related to arousal | *T* score (mean = 50; SD = 10); higher scores indicate worse anxiety. |
| PROMIS®  Fatigue | 4-item Short Form that assesses fatigue | *T* score (mean = 50; SD = 10); higher scores indicate worse fatigue. |
| PROMIS®  Anger | 4-item Short Form that assesses angry mood (irritability, frustration), negative social cognitions (interpersonal sensitivity, envy, disagreeableness), and efforts to control anger. | *T* score (mean = 50; SD = 10); higher scores indicate worse anger. |
| PROMIS®  Pain Interference | 4-item Short Form that assesses pain interference. | *T* score (mean = 50; SD = 10); higher scores indicate worse pain interference. |

**Table S2.** Student wellness interview guide. Y: Yes, N: No.

| 1. Please describe your understanding of this study.    1. Your understanding of the app?    2. Your understanding of the wellness coaching?    3. Your understanding of combining the two?    4. Are you glad to have participated?    5. Did it reduce stress? Increase stress?    6. What aspects of the study did you find helpful to support your mental health? |
| --- |
| 1. Approximately how often did you use the app?    1. Never (other than onboarding, doing surveys), daily, weekly, at end?    2. Which components of the app did you use?       1. State each of the Positive Activities – Y or N?       2. Mood – did you enter on daily, weekly basis ­– Y or N?       3. Fitbit (graphs) – did you view them? How often? |
| 1. What were your expectations of the study?    1. The app?    2. The wellness coaching?    3. Did it meet or not meet your expectations? |
| 1. Overall, tell me about your experience with:    1. Using the app?    2. Participating in wellness coaching?    3. Did you prefer one over the other? Or did the two complement one another by reinforcing certain concepts or topics? |
| 1. Some additional probing questions that you (the moderator) could consider inquiring about depending on respondent’s interaction with you:    1. Were there any facilitators of app use [or coaching] that you can think of?    2. Were there any barriers of app use [or coaching] that you can think of?    3. Were the Daily Mood prompts helpful? Y or N, if helpful, please describe how so    4. Did the app create any routine(s) or new habit(s)?    5. Did the app promote expressive opportunities – through rating your mood, communicating feelings verbally.    6. Did the app impact:  - Your self-awareness; for example, were you able to recognize and differentiate different states of emotions) - Your emotional control - Checking in with oneself - Your ability to take your mind off any negative feelings? - Stress levels – reduce or increase? |
| 1. Which features did you use most often and why? |
| 1. Which features did you like the most and why? |
| 1. Which features did you find the most helpful and why? |
| 1. Which features did you use the least often and why? |
| 1. Which features did you like the least and why? |
| 1. Which features did you find the least helpful and why? |
| 1. Did anything interfere with you using the app? Participating in wellness coaching? 2. What would make you more likely to use the app? |
| 1. Do you have any suggestions or recommendations to improve the app? The wellness coaching? The overall experience of participating in a similar study? |
| 1. Would you participate in a similar study? For example, what if we designed a study where you were randomized to receive coaching versus no coaching in addition to the app? |
| 1. Have you ever used anything like this before; if yes, please describe it? |
| 1. Do you have any recommendations or suggestions to increase the likelihood of someone else using the app in the future? |
| 1. Is there anything else you would like to share about your experience with the study? The app? The wellness coaching? |
| 1. If we could add personalization, would you like notifications based on your prior day’s steps, mood, or sleep, activity used/not used?  - What is the frequency of notifications that seems appropriate without causing too much burden or where you may start ignoring them – e.g., once a day, once a week, twice a week? |

**Table S3.** Student wellness interview codebook. Purpose: Explore the feasibility of using wellness coaching and Roadmap app together, and ways to improve their interactions to better support student wellness and mental health

| **Code Name** | **Code** | **Description** | **Examples** |
| --- | --- | --- | --- |
| Study General Feedback | GEN-FEEDBACK | Student expresses general feedback about the study | “I really like it. I think it was really cool to have these tools to track my wellness and try to use the tools to improve mindfulness.” |
| Roadmap General Feedback | RM-GEN-FEEDBACK | Student expresses general feedback about Roadmap | “I was going to say I like how it’s set up and everything.” |
| Wellness Coaching General Feedback | COACH-GEN-FEEDBACK | Student expresses general feedback about wellness coaching | “Wellness coaching I feel like is all encompassing. It helps me with any aspect, I feel like. I’m able to talk through things more. A lot of times what I really need help with is literally just having someone to bounce stuff off of. She kind of reflects what I say back and processes through things.” |
| No Preference for Coaching or Roadmap | PREF-NONE | Student has no preference between Roadmap app and wellness coaching | “I think I see them as equal. It’s nice to have someone to talk to, but then it’s also nice to be able to go on your phone and put in things to help you throughout the day.” |
| Coaching Preference | PREF-COACH | Student prefers wellness coaching over Roadmap app | “The app in general I was not a big fan of. … Well, I love wellness coaching.” |
| Roadmap Preference | PREF-RM | Student prefers Roadmap app over wellness coaching | “I preferred the app over wellness coaching. The app is obviously much less of a time commitment in terms of blocking out an entire hour to schedule a wellness session, whereas the app, it’s sort of on your own time. The app is also a little bit more self directed which I enjoy.” |
| Study Increased Stress | INC-STRESS | Student reports that participation in study increased feelings of stress | “I think it's a very, very complex thing, because at the beginning it improved… Sorry, not improved, it increased my stress a little bit because I was literally aware of how low my daily energy consumption is. I tried to improve or add more physical activities to it to make me meet the goals, like the number of steps I took and the number of calories I burned.” |
| Study Decreased Stress | DEC-STRESS | Student reports that participations in study decreased feelings of stress | “Participating definitely reduced stress levels, for me at least. Working out and being physically active really helps reduce my stress levels. Having the Fitbit and the Roadmap to monitor my sleep levels and my activity levels definitely reduced my stress.” |
| Study Did Not Affect Stress | NE-STRESS | Students reports that participation in study had no impact on stress levels | “It stayed the same” |
| Study Energy Requirement | ENERGY-REQ | Student reports that participation in study required excessive amounts of energy to partake in | “Doubling that for the app, it required more energy and time” |
| Used Roadmap Frequently | RM-USE-FREQ | Student mentions frequent (multiple times per week) use of Roadmap app | “If I had to put a number to it, I would probably say three out of seven days of the week. Maybe four.” |
| Used Roadmap Infrequently | RM-USE-INFREQ | Student mentions infrequent (multiple times per month) use of Roadmap app | “I used it in the beginning for about two weeks. I didn’t even use it that much then.” |
| Used Roadmap Rarely | RM-USE-RARE | Student mentions rare (only a few times throughout the study) use of Roadmap app | “‘Could you estimate approximately how much you did use the app?’  ‘Once or twice.’” |
| Roadmap Use Declined Over Time | RM-USE-DEC | Student mentions that their usage of the Roadmap app declined over time | “I used it in the beginning for about two weeks. … Towards the end, I didn’t like using it at all.”  “At first I definitely used it more, probably once a day. Then just as I had a lot on my schedule, probably more like once a week.” |
| Participation Positive Feedback | POS-PART | Student expresses positive feedback to participating in the study | “Overall, the study was a pretty good experience” |
| Participation Neutral Feedback | NEU-PART | Student expresses neutral feedback to participating in the study | “I would say neutral. I’m not glad, I’m not upset about it.” |
| App Design Positive Feedback | POS-DES | Student expresses positive feedback to the design of the app | “No. I found it really easy to use.” |
| App Design Negative Feedback | NEG-DES | Student expresses negative feedback to the design of the app | “I always speak to the user flow. When things were not very clear for me, for example scheduling activities. The flow of that did not make sense.” |
| Positive Activity Positive Feedback | POS-ACT | Student expresses positive feedback to positive psychology activities | “I like the Gratitude Journal and Savoring. Savoring was nice.” |
| Positive Activity Negative Feedback | NEG-ACT | Student expresses negative feedback to positive psychology activities | “I used it in the beginning. Then I felt like this was just like another thing to do.” |
| Roadmap Met Expectations | POS-RM-EXP | Student mentions how their expectations of the Roadmap app going into the study were met or exceeded | “They’re definitely met, yes. They’re exceeded, honestly.” |
| Roadmap Did Not Meet Expectations | NEG-RM-EXP | Student mentions how their expectations of the Roadmap app going into the study were not met | “‘Do you think those expectations were met or not?’  ‘No’ (laughs).” |
| Coaching Met Expectations | POS-COACH-EXP | Student mentions how expectations of wellness coaching were met or exceeded | “It actually worked out better than I expected. I developed a personal connection with Marsha. I’m very grateful for that, and I enjoyed it a lot. She really offered a lot of really good suggestions to me, and yes, I really appreciate that. Yes.” |
| Study Met Expectations | POS-STUDY-EXP | Student mentions how expectations of study were met or exceeded | “I think it was better than I expected, if anything.” |
| Study Did Not Meet Expectations | NEG-STUDY-EXP | Student mentions how expectations the general study were not met | “To be honest, not really. I know the whole purpose of the study was to help students tackle their issues. I don’t know if I am a good indicator of that because I felt like I knew what I had to do, but I just felt too lazy to do it sometimes. I don’t know if that helps or not.” |
| Wearable Fitness Tracker Positive Feedback | POS-WT-FEEDBACK | Student expresses positive feedback towards the FitBit watch or other wearable fitness trackers | “What I enjoyed most about the study was getting the actual Fitbit and using that to monitor my steps per day, my heart rate when I work out, how many calories I’m burning with my workout.” |
| Wearable Fitness Tracker Negative Feedback | NEG-WT-FEEDBACK | Student expresses negative feedback towards the FitBit watch or other wearable fitness trackers | “I think there is only one thing, it’s the notification of Fitbit is weird. It doesn’t … The things that they push are not quite related to what I have done, but it’s more advertisement.” |
| Graphs Tab Positive Feedback | POS-GRAPH | Student expresses positive feedback to the section of the Roadmap app that contains graphs of personal data (e.g. sleep, steps, heart rate) | “Keeping track of my sleep was very, very useful to me. Most things pertaining to my physical health, I would say.” |
| Graphs Tab Negative Feedback | NEG-GRAPH | Student expresses negative feedback to the section of the Roadmap app that contains graphs of personal data (sleep, steps, heart rate) | “You have all this data, it doesn’t necessarily change my behaviors. I’d still just do the same amount of walking and attend the same classes.” |
| Chat Forum Positive Feedback | POS-CHAT | Student expresses positive feedback about the Roadmap Chat Forum | “Another thing is that I think the forum overall is pretty good.” |
| Chat Forum Negative Feedback | NEG-CHAT | Student expresses negative feedback about the Roadmap Chat Forum | “I never used the chat forum. At all. I also have no interest in using the chat forum.” |
| Facilitators of Roadmap Usage | FACIL-APP | Student mentions specific facilitators that encouraged Roadmap usage | “The notifications on my phone is a facilitator, if that counts.” |
| Barriers of Roadmap Usage | BARR-APP | Student mentions specific barriers that prevented Roadmap usage | “I think the biggest barrier to the app was – Honestly, there weren’t many. Part of it was just remembering it was there.” |
| Barriers of Coaching | BARR-COACH | Student mentions specific barriers that prevented going to Wellness Coaching | “I wanted to go, but I was teaching and I was doing several other things. I scheduled an appointment thrice, and I had to miss every time, or reschedule it. … Ultimately, we had to cancel because I just couldn’t find the time.” |
| Daily Mood Notification Positive Feedback | POS-MOOD | Student expresses positive feedback to the daily mood notification | “A hundred percent, yes. Notifications were very helpful.” |
| Daily Mood Notification Negative Feedback | NEG-MOOD | Student expressed negative feedback to the daily mood notification | “The reason I also did not like the mood thing, it would be like, enter what your daily mood was or whatever the question was. I basically always put a seven. Your mood changes throughout the day. … I would prefer as a user, I’d be interested to see how much mood changes throughout the day. To be able to identify what activities or what thing I’m doing that are causing either a really good mood or a bad mood. That would make sense to me versus what was your mood overall in the day.” |
| Notification Modification Suggestions | MOD-NOTIF | Student makes suggestion about changing the notification system of the Roadmap app | “I just wanted to change it to be like a half hour earlier” |
| New Habits from Roadmap | RM-HABIT | Student develops new habits or routines from Roadmap app | “I would make a habit to be more – One of the features, it’s a little piggy band thing, when something that you remember occurs in your life, you type it in, put it in the piggy bank, and then you break it open. I’d say that that definitely had the effect of influencing me to use the app more.” |
| Roadmap Increased Expressive Opportunities | INC-EXPR-OPP | Student mentions that the Roadmap app increased their expressive opportunities | “Yes, for sure. A lot of prompts have the option to write, so it just helps to verbalize what I’m actually feeling in the moment, or put the picture, or anything that I would want to input in the app.” |
| Roadmap Had No Effect on Expressive Opportunities | NE-EXPR-OPP | Student mentions that the Roadmap app had no effect on their expressive opportunities | “I don’t necessarily think that it made me communicate ‘better,’ I think it was another pathway to do that communication. I don’t know if it was better or worse, though, I can’t really say.” |
| Roadmap Increased Self-Awareness and/or Emotional Control | SELF-EMOT | Student mentions how the Roadmap app increased their level of self-awareness and/or emotional control | “I do think it made me more aware of how I was feeling, especially if things were more negative, and try to turn it into a positive light. It did make me more self aware” |
| Roadmap Had No Impact on Self-Awareness and/or Emotional Control | N-SELF-EMOT | Student mentions how the Roadmap app had no impact on their level of self-awareness and/or emotional control | “‘Do you think the app impacted your self-awareness at all?’  ‘No.’” |
| Roadmap Decreased Negative Feelings | DEC-NEG-FEEL | Student reports that the Roadmap app decreased negative feelings | “‘It helped you take your mind off of negative feelings?’  ‘Yes. Definitely.’” |
| Roadmap Had No Impact on Negative Feelings | NE-NEG-FEEL | Student reports that the Roadmap app had no impact on negative feelings | “‘Did it help take your mind off of negative feelings?’  ‘No. Tracking my physical health helped.’” |
| Most Used Feature | M-USE-FEAT | Student describes one or two of their most used features | “Definitely the sleep monitoring part I used the most. Besides that, the next one would be the daily mood survey, ranking my mood every day and looking at that progress throughout the study.” |
| Least Used Feature | L-USE-FEAT | Student describes one or two of their least used features | “The signature strengths was definitely the one that I probably used the least.”  “I think, love letter, I only did that once.” |
| Most Liked Feature | FAV-FEAT | Student describes one or two of their most liked features and why they favored said features | “I like the gratitude and the savoring the most. Those are the things I pay attention to the most.” |
| Least Liked Feature | L-FAV-FEAT | Student describes one or two of their least liked features and why they disliked said features | “I least liked the love letter instructions. …If I wanted to give someone a love letter, I’d give it to them. I wouldn’t put it in the app.” |
| Modifications to Promote More App Usage | MOD-MORE-APP-USE | Student provides suggestions to modify the Roadmap app in a way that promotes increased app usage | “I think simplifying things. When I checked the instructions for each little tool, it had this very long structured way that you were supposed to use the app, and I wan’t like, ‘that’s not quite how I would use it.’” |
| Willingness to Participate in Similar Study | POS-NEW-STUDY | Student expresses willingness to participate in a similar study | “Yes, I would participate.” |
| Unwillingness to Participate in Similar Study | NEG-NEW-STUDY | Student does not express willingness to participate in a similar study | “No, I don’t think I would participate if it were randomized. I think I’d want to be able to have the choice.” |
| Roadmap Complement | RM-COMP | Student mentions an external activity that serves as a complement to the Roadmap app | “I am one of those people, even though I have a lot of friends, I’d rather talk to ChatGPT rather than some of my friends, because I do not want to put burden on others.” |
| Roadmap Interference | RM-INT | Student mentions an external activity that serves as an interference to the Roadmap app | “I already do the gratitude journal for instance. Me, doubling that for the app, it required more energy and time. I prefer to write in my journal because I use these cute, fun sticky notes.” |
| No Existing Roadmap Substitute | NO-EXIST-RM-SUB | Student mentions that there is no existing substitute for the Roadmap app | “No, I have not used a similar app at all. This is a very unique app.” |
| Existing Roadmap Substitute | EXIST-RM-SUB | Student mentions that there is an existing substitute for the Roadmap app | “Actually, I think it’s very similar to some other apps that I have seen before, like Fitness. I think it’s something that iPhone had, and also some other apps. … There is a Chinese one called Xiaomi.” |
| Personalized Notifications Positive Feedback | POS-PERS-NOTIF | Student provides positive feedback to the possibility of personalized notifications | “I go to bed really early, and you couldn’t change the notification time. … I just wanted to change it to be like a half hour earlier” |
| Frequency of Notifications Feedback | FREQ-NOTIF | Student provides general feedback to the frequency of the notifications | “I’d like to be able to customize it. I’d like to be able to make ‘engaging with beauty’ remind me every two days, and then ‘random acts of kindness’ every seven. I’d want to be able to change that.” |
| Interpersonal Relations Positive Feedback | POS-IR | Student provides positive feedback to interpersonal connections fostered by Roadmapp app or wellness coaching | “The kindness thing. Yes, I like that one. I read that one a lot. It makes me feel better when I’m reading people record the happiness and just tiny kindnesses they received in their lives. Yes, it makes me feel better, feel good, feel happy.” |
| Coaching and Roadmap Compatibility Feedback | COMP-RM-COACH | Student provides feedback about how Roadmap and Wellness coaching work together or as separate entities | “It felt like three different things to me instead of connecting the three. It was like, let’s see what a Fitbit does, wellness coaching, and an app. Versus not thinking about how they’re incorporated and how they build off from each other, if that makes sense” |

**Table S4.** Coaching sessions codebook.

| Codename: | Code: | Description: | Examples from the coaches’ notes: |
| --- | --- | --- | --- |
| Potential Solutions | POT-SOL | Coaches mention potential solutions to help the students with any of their issues | “Practice RAIN (Recognizing, Allowing, Investigating, and Nurturing) self-compassion exercises” |
| Sleep Issues | SLEEP | Student mentions issues related to sleep | “Mom encouraged her to seek some support for challenges she is experiencing around sleep and motivation. Having trouble falling asleep and oversleeping. Missing class.” |
| Anxiety Issues | ANXIETY | Student mentions issues related to anxiety | “Depression and anxiety leads to feelings of fatigue, brain fog, difficulty focusing etc. Unsure cause/effect. If mental health is impacting school, or difficulties with school led to anxiety and depression.” |
| Body Image Issues | BOD-IMG | Student mentions issues related to body image | “Fat phobic with her body/self image/noticing how it feels/needs to cover her stomach even when alone” |
| Academic Issues | ACA-ISS | Student mentions issues regarding academics or school life | “She has a lot of perfectionist tendencies and really beat herself up for dropping the class, feeling like a failure” |
| Social Issues | SOC-ISS | Student mentions issues concerning social life or relationships | “Shared that while connection and happiness are two of the biggest values for him, he finds it hard to build new connections at UM (University of Michigan), unsure what to ask people to do or what would bring him joy.” |
| Time Management Issues | TM-ISS | Student mentions issues with time management | “Balancing being in school and being a mom, along with her health and her marriage has been a lot to juggle.” |
| Miscellaneous Issues | MISC-ISS | Student mentions any other issues they are struggling with | “She has a lot of things going on right now including complex PTSD (Post Traumatic Stress Disorder) and possibly fibromyalgia and ADHD (Attention-deficit/hyperactivity disorder).” |
| Desire to Improve Health | DES-IMP-HEALTH | Student indicates desire to improve mental and/or physical health | “She wants a better physical connection with body and better mental health.” |
| Improvements to Health | IMP-HEALTH | Students indicate that their health has improved during the course of the study | “Sleep has been improving slightly–able to fall asleep more easily and seeing improved trend on Fitbit app. Not perfect yet but will continue to work on it” |

**Table S5.** Pre- and Post-study PROMIS® outcomes. SE: standard error.

| **Complete surveys** | **Mean (SE)** | ***P*-value** | **Adjusted *P*-value** |
| --- | --- | --- | --- |
| Ability to participate in social roles and activities | 5.32 (2.39) | 0.035 | 0.096 |
| Anxiety | −9.12 (2.71) | 0.002 | 0.04 |
| Cognitive function | 3.94 (2.45) | 0.118 | 0.161 |
| Depression | −9.01 (2.98) | 0.005 | 0.043 |
| Fatigue | −8.91 (3.48) | 0.016 | 0.049 |
| Pain interference | −1.43 (2.02) | 0.484 | 0.484 |
| Physical function | 2.38 (1.21) | 0.056 | 0.114 |
| Sleep disturbance | −2.7 (1.87) | 0.156 | 0.187 |
| Anger | −9.03 (3.48) | 0.015 | 0.049 |
| Emotional support | 4.57 (2.59) | 0.088 | 0.129 |
| Companionship | 3.59 (2.49) | 0.158 | 0.187 |
| Global physical health | 4.24 (2.41) | 0.0881 | 0.129 |
| Global mental health | 6.36 (2.4) | 0.0118 | 0.049 |
| Informational support | 2.63 (2.54) | 0.3088 | 0.326 |
| Meaning and purpose | 4.89 (3.72) | 0.1989 | 0.222 |
| Positive affect | 10.29 (3.52) | 0.0072 | 0.046 |
| Satisfaction roles and activities | 4.54 (2.47) | 0.0750 | 0.129 |
| Sleep-related impairment | −6.99 (3.58) | 0.0599 | 0.114 |
| Social isolation | −7.42 (3.53) | 0.0465 | 0.110 |

**Table S6.** Joint table of quantitative and qualitative findings. All codes are defined in Tables S3 and S4.

| **Theme** | **Quantitative data** | **Qualitative data** | **Merged data** |
| --- | --- | --- | --- |
| Roadmap app and wellness coaching synergy | Timestamps of overall app use (mood entry, positive activity use, chat forum post, chat forum view)    Number (#) of wellness coaching sessions scheduled | Code: COMP-RM-COACH    “*For me, the app was a nice place to reflect on how I’d been feeling and then bring that to the wellness coaching. I was more prepared with stuff that I knew to work on.”*    Code: GEN-FEEDBACK    “*It was a really great study, honestly. I feel like it did really help altogether. All the pieces, I felt like, fit together with using the Fitbit, the app, and the wellness coaching. It was definitely a good study and I think it’s good for college students and probably a lot more people out there to just have that positivity brought into their life and focus on the positive things.*” | Analysis of app usage timestamps (Figure 3) and the recorded number of scheduled wellness coaching sessions (mean = 2, range = 1–5) combined with interview data revealed patterns of students engaging with both intervention components.    Nearly all participants stated “*Yes”* to *“are you glad to have participated?”* One participant replied, *“neutral, neither glad or upset.”* |
| Perceived impact of intervention on mental health | PROMIS®  Assessments Pre- and Post-Study | Code: GEN-FEEDBACK    *“Wellness coaching, I feel like is all encompassing. It helps me with any aspect, I feel like. I’m able to talk through things more. A lot of times what I really need help with is literally just having someone to bounce stuff off of. She kind of reflects what I say back and processes through things. The app, I felt like, was more about creating self-healthy habits. I guess that’s just in general how I would sum it up. Healthy habits, mentally. More on the mental side for that one. “* | Analysis of pre- and post-study PROMIS®  assessments (Figure 3) and longitudinal mood data (Figure 6) combined with interview data suggests overall positive impact on mental health outcomes (i.e., improved global mental health and reduced depression, anxiety, fatigue, and mood scores) |
| Positive activity (PA) use | Timestamps of PA use | Code: RM-HABIT    *“One of the features, it’s a little piggy bank thing, when something that you want to remember occurs in your life, you type it in, put it in the piggy bank, and then you can break it open. I’d say that that definitely had the effect of influencing me to use the app more.”*  Code: POS-MOOD  *“The one thing that made me want to use it more was, of course, I loved the daily survey, and I also enjoyed, also reading what other people had written [Chat Forum posts/view], of what they had, as well. I thought that was also a pretty cool feature. That made me get where I’m getting to a little bit more.”* | The timestamps of PA use (Figure 4) provided useful data about patterns of use. According to users, the app assisted with habit formation. This was a new code that emerged from the interviews (RM-HABIT). Several users commented on the community aspect of the Chat Forum and enabled users to post and view comments. Also, supported in the above quote about the intervention’s impact on overall mental health. |
| Areas of improvement | Timestamps of overall app use    Number of coaching sessions | Code: BARR-COACH    *“I think the wellness coaching went really well, but it was challenging to schedule through the online portal.”*    Code: MOD-MORE-APP-USE    *“I would say maybe having a notification feature. There’s notification for the daily moods, but there’s not really any notifications just to use any of the other features.”*    Code: NEG-DES    “*I did notice the app glitches a lot. It will force quit and kick me out often…*” | Merged analysis of app usage patterns and coaching session data suggests that both app engagement and participation in wellness coaching declined over time, with users averaging only two coaching sessions (range: 1–5) and five students never attending a session. Qualitative feedback highlighted several barriers to sustained involvement, including complex or overly structured app instructions, technical glitches, lack of customized notifications, and difficulties scheduling coaching sessions through the online portal. Time constraints were also a common obstacle, highlighting the need for more user-friendly and accessible intervention features. |

References

1. Rozwadowski M, Dittakavi M, Mazzoli A, Hassett AL, Braun T, Barton DL, et al. Promoting health and well-being through mobile health technology (Roadmap 2.0) in family caregivers and patients undergoing hematopoietic stem cell transplantation: protocol for the development of a mobile randomized controlled trial. JMIR Res Protoc. 2020; 9(9):e19288. doi: 10.2196/19288
