# Supplementary figures and images for: Blended mobile health and wellness coaching enhances student engagement in mental health care
Source: Acad Ment Health Well Being. Author manuscript; Available in PMC 2026 Jun 26. (PMC13298186; doi:10.20935/mhealthwellb8298)

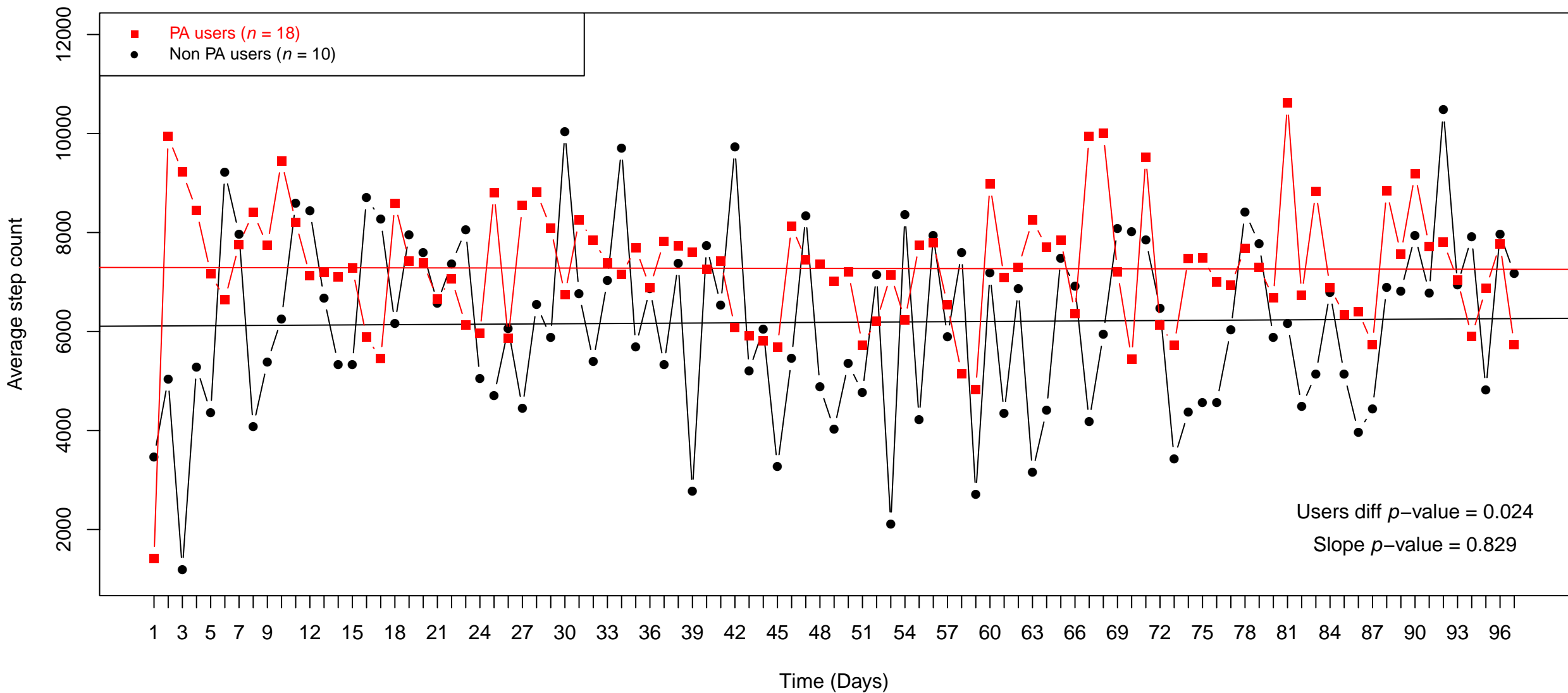

Supplement: Supplemental Figure 1 [file NIHMS2181429-supplement-Supplemental_Figure_1.pdf]

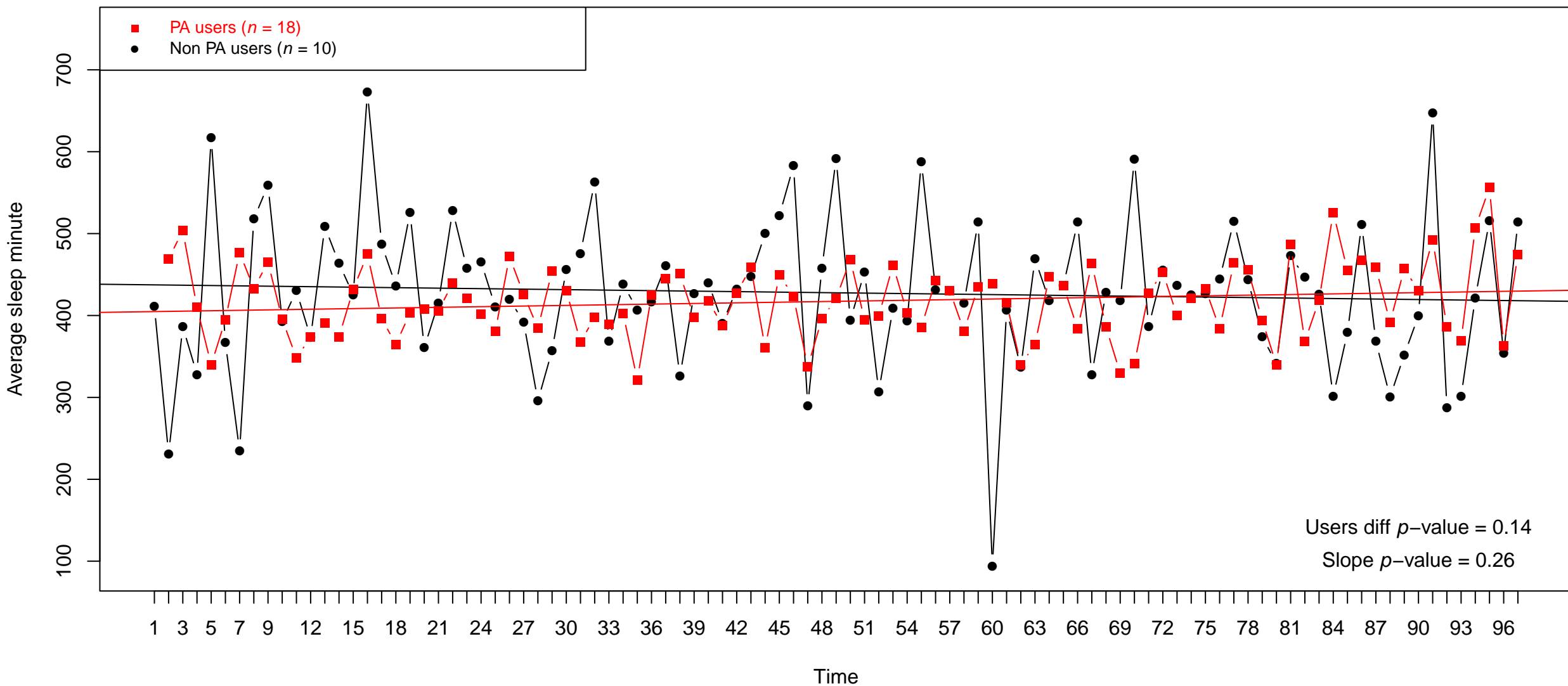

Supplement: Supplemental Figure 2 [file NIHMS2181429-supplement-Supplemental_Figure_2.pdf]

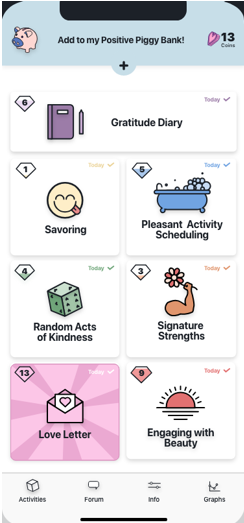

Supplement: Supplemental Figure 3 [file NIHMS2181429-supplement-Supplemental_Figure_3.png]
